# BCR::ABL1 tyrosine kinase inhibitors induce ribosome collisions to activate ZAK-dependent ribotoxic stress and apoptosis in chronic myeloid leukemia

**DOI:** 10.1038/s41375-026-02916-3

**Published:** 2026-03-30

**Authors:** Jumin Park, Soo-Hyun Kim, Jongmin Park, Heeju Park, Hongtae Kim, Dong-Wook Kim, Chunghun Lim

**Affiliations:** 1 Department of Biological Sciences, Korea Advanced Institute of Science and Technology, Daejeon, Republic of Korea; 2 Leukemia Omics Research Institute, Eulji University, Uijeongbu‑si, Gyeonggi‑Do Republic of Korea; 3 Department of Biological Sciences, Ulsan National Institute of Science and Technology, Ulsan, Republic of Korea; 4 Hematology Department, Eulji Medical Center, Eulji University, Uijeongbu‑si, Gyeonggi‑Do Republic of Korea; 5 Graduate School of Stem Cell and Regenerative Biology, Korea Advanced Institute of Science and Technology, Daejeon, Republic of Korea

**Keywords:** Chronic myeloid leukaemia, Oncogenes, Cell signalling, Disease genetics

## Abstract

Ribosome collisions act as molecular sensors of cellular stress, yet their role in disease physiology remains unclear. Here, we demonstrate that inhibition of the oncogenic kinase BCR::ABL1 in chronic myeloid leukemia (CML) cells induces ribosome collisions and activates the ribotoxic stress response (RSR). Clinical analyses revealed that CML progression from the chronic phase to the aggressive blast phase correlated with elevated expression of the RSR-initiating kinase ZAK. Although ZAK sustained CML cell proliferation by promoting AKT activity, loss of *ZAK* function paradoxically reduced the cytotoxic effects of BCR::ABL1 inhibitors. Mechanistically, BCR::ABL1 inhibition promoted phosphorylation of eukaryotic translation elongation factor 2 (EEF2) via the mTOR-EEF2K pathway, slowed translation elongation, and generated nuclease-resistant collided ribosomes that triggered ZAK-dependent p38 activation and apoptosis. Furthermore, pharmacological modulation of translation flux fine-tuned the efficacy of BCR::ABL1 inhibitors, including in primary patient cells. These findings define a ribosome-based stress pathway crucial for CML apoptosis and highlight ZAK-dependent RSR as a therapeutic vulnerability.

## Introduction

Ribosomes are essential molecular machines that translate mRNA-encoded genetic information into functional polypeptides. However, translating ribosomes can pause on mRNAs under various translation environments. For instance, ribosome stalling and collisions occur during aberrant translation events [e.g., poly(A)-tail translation due to splicing errors or stop-codon readthrough], thereby activating the ribosome-associated quality control (RQC) pathway. Co-translational RQC disassembles collided ribosomes for recycling and directs translation intermediates (i.e., prematurely terminated polypeptides) for proteasomal degradation [1–7]. Ribosome collisions can also arise more broadly under translation stress conditions, initiating a ribotoxic stress response (RSR). Known RSR inducers include ribotoxins (e.g., ricin), translation inhibitors (e.g., anisomycin), cellular energy depletion (e.g., amino acid starvation), and environmental stressors (e.g., UV radiation, oxidative stress), all of which disrupt ribosome function, damage RNA, or impair translation processes [8–13]. The downstream RSR operates at multiple levels, from transcriptional regulation to cell fate decisions [4, 14–16]. Accordingly, the dynamic behavior of translating ribosomes serves as a critical cue for maintaining proteostasis and eliciting adaptive cellular responses.

A hallmark of the RSR pathway is the activation of dedicated mitogen-activated protein kinase (MAPK) signaling upon translation stress. Ribosome pausing activates the specific MAP3 kinase ZAK (MAP3K20; also known as MLTK/MLK7), which initiates the kinase cascade leading to the stress-activated protein kinases (SAPKs; JNK and p38) and amplifies the RSR [12, 13]. RSR activation results in diverse cellular outcomes (e.g., inflammation, cell cycle arrest, and apoptosis) depending on the severity and persistence of stress signals [11, 17–23]. Recent findings suggest that ZAK functions as a molecular sensor that distinguishes between tolerable and lethal levels of ribotoxic stress, thereby influencing cell fate decisions (e.g., cell survival vs. programmed cell death) [12, 13]. The functional role of ZAK extends beyond translation stress, as it has been implicated in metabolism, immune signaling, and neurodegeneration [24–27]. These ribosome-associated pathways emphasize that the ribosome serves not only as a genetic decoder but also as an active regulator of cellular physiology.

Not surprisingly, translational dysregulation has been implicated in various human diseases, including cancer [28–31]. An illustrative example is the molecular pathogenesis of the BCR::ABL1 tyrosine kinase, an oncogenic driver in chronic myeloid leukemia (CML). Reciprocal translocation between chromosomes 9 and 22 generates the Philadelphia chromosome, which is present in CML patients and encodes a constitutively active tyrosine kinase designated as the BCR::ABL1 oncoprotein [32, 33]. The BCR::ABL1 kinase modulates multiple signaling pathways involved in proliferation, survival, and metabolic adaptation [34–36]. In particular, BCR::ABL1 activates the PI3K-AKT-mTOR pathway to drive protein synthesis via downstream effectors (e.g., ribosomal protein S6 and 4E-BP1), supporting the proliferation and malignant behavior of CML cells [37–39]. On the other hand, tyrosine kinase inhibitors (TKIs) specifically targeting the pathogenic BCR::ABL1 fusion protein have been developed for CML treatment. Imatinib (also known as Gleevec) is a first-generation TKI that specifically binds to the ATP-binding site of BCR::ABL1 in its inactive state, thereby inhibiting the kinase activity and serving as an effective therapeutic agent for CML [40, 41].

Some CML patients, however, develop resistance to TKIs through various molecular mechanisms, including secondary mutations in the BCR::ABL1 kinase domain or activation of alternative survival pathways such as mTOR signaling [42–44]. In fact, mTOR inhibitors (e.g., rapamycin) enhance the anti-leukemic effects of BCR::ABL1 TKIs by inhibiting protein synthesis and inducing apoptosis, even in TKI-resistant CML cells [43, 45–48]. The translation elongation inhibitor homoharringtonine (HHT) has been primarily utilized as an anticancer agent and has gained attention due to its effectiveness in the treatment of hematological malignancies such as CML [49–51]. Consequently, it was FDA-approved in 2012 for the treatment of TKI-resistant CML patients [52, 53]. These previous findings potentially link oncogenic BCR::ABL1 signaling to ribosome activity with clinical relevance. We thus explored whether pathogenic changes in translation dynamics and relevant pathways are associated with CML biology and its treatment.

## Methods

### Cell lines

CCRF-CEM, MOLT-4, and Jurkat cell lines were purchased from the Korean Cell Line Bank. HL-60, MOLM13, MOLM14, THP-1, KU812, K562, and K562^IMres^ cell lines have been described previously [54]. A *ZAK*-deleted K562 cell line (*ZAK*^KO^) was generated by *ZAK* sgRNA-expressing lentiviral transduction in the presence of polybrene (8 µg/mL). At 48 h post-infection, transduced cells were selected with puromycin (2 µg/mL) for 14 days, then single-cell-sorted to establish independent clonal lines.

### CML patient samples

Unpaired samples of bone marrow (BM) or peripheral blood (PB) were obtained from 89 CML patients (CP = 48; AP = 1; BP = 52). Serial samples of BM or PB were obtained from 10 CML patients during their transitions between distinct pathogenic stages (complete hematologic response = 14; no evidence of leukemia = 3, CP = 5, BP = 15). Mononuclear cells (MNCs) were isolated by density gradient centrifugation using Ficoll-Paque (GE Healthcare, STEMCELL). Samples were frozen in fetal bovine serum (FBS; Sigma-Aldrich) with 10% dimethyl sulfoxide (DMSO; Sigma-Aldrich), stored in liquid nitrogen, and thawed before subsequent analyses. All human samples were obtained from the Korea Leukemia Bank. The protocol was approved by the Institutional Review Board. Patient consent was obtained in accordance with the Declaration of Helsinki.

### DNA constructs and siRNAs

pcDNA4/TO/Strep-HA-ZAK alpha (Addgene plasmid #141193) and pcDNA4/TO/Strep-HA-ZAK alpha_K45A (Addgene plasmid #141194) were gifts from Simon Bekker-Jensen [12]. ZAK alpha_F368C and ZAK alpha_K45AF368C cDNAs were generated by PCR-based mutagenesis from pcDNA4/TO/Strep-HA-ZAK alpha and subcloned into the same backbone vector. P210 pcDNA3 (Addgene plasmid #27481) was a gift from Warren Pear [55]. pLentiPGK Puro DEST p38KTRClover (Addgene plasmid #59152) was a gift from Markus Covert [56]. The oligonucleotide pair encoding a *ZAK*-specific sgRNA (5′-ATGGATATCACAGGACAAGG-3′) was synthesized (Macrogen) and cloned into lentiCRISPR v2 (Addgene plasmid #52961; a gift from Feng Zhang) [57] to produce recombinant lentiviruses for genomic *ZAK* deletion. Gene-specific siRNAs were synthesized (Genolution and Bioneer) as listed in Supplementary Table S1.

### Chemicals

Anisomycin (Sigma-Aldrich), asciminib (MedChemExpress), cycloheximide (CHX; Sigma-Aldrich), harringtonine (Cayman chemical), homoharringtonine (Sigma-Aldrich), imatinib mesylate (Selleckchem), MG132 (APExBIO), MK-2206 dihydrochloride (MedChemExpress), nelfinavir (APExBIO), SB203580 (MedChemExpress), SC79 (MedChemExpress), sorafenib (APExBIO), SP600125 (MedChemExpress), Torin 1 (Selleckchem), and 4EGI-1 (MedChemExpress) were dissolved in dimethyl sulfoxide (DMSO; Sigma-Aldrich) and stored at −20 °C before use. Puromycin dihydrochloride (Sigma-Aldrich) was dissolved in phosphate-buffered saline (PBS) and stored at −20 °C before use.

### Cell cultures, transfection, proliferation, and apoptosis assays

Immortalized cell lines and patient-derived BMMNC were cultured in RPMI 1640 medium (GenDEPOT; Sigma-Aldrich; SOL Bio) with 10% FBS (Gibco) at 37 °C in a 5% CO_2_ atmosphere. The primary BMMNC cultures were further supplemented with 1% penicillin/streptomycin (Gibco). Transient transfection was performed using the Neon Transfection System (Invitrogen) or Lipofectamine 2000 (Invitrogen) according to the manufacturer’s instructions. For electroporation, cells were transfected with plasmid DNA or siRNA using a microporator (1450 V/15 ms/2 pulses for K562 and KU812 cells; 1350 V/10 ms/3 pulses for Jurkat cells; 1750 V/20 ms/1 pulse for BMMNC) and incubated in the culture media for 48 h before harvest. For liposome-based transfection, cells were incubated with the DNA-lipid complex for 48 h before harvest. For cell proliferation assay, siRNA-transfected cells were split into 60-mm culture dishes (5000 cells per dish) at 48 h posttransfection. Each well was harvested at the indicated time point, and the total number of cells was counted using LUNA-II automated cell counter (Logos Biosystems). For apoptosis assay, cells were harvested by centrifugation at 2000 rpm for 3 min at room temperature and washed twice with PBS. Apoptotic cells were detected using the FITC Annexin V Apoptosis Detection Kit I according to the manufacturer’s instructions (BD Bioscience). The percentage of apoptotic cells was assessed in 10,000–50,000 cells by flow cytometry (LSRFortessa, BD Biosciences) and quantified using FlowJo software.

### Immunoblotting analysis

Cells were harvested, washed once with PBS, and lysed in a RIPA buffer [50 mM Tris-Cl pH 8.0, 150 mM NaCl, 1% NP-40 (v/v), 0.5% sodium deoxycholate, 0.1% sodium dodecyl sulfate (SDS)] containing 1 mM phenylmethylsulfonyl fluoride (PMSF) and 1 mM dithiothreitol (DTT) at 4 °C for 15 min. For puromycin labeling of nascent polypeptides, cells were incubated with 10 µg/ml puromycin dihydrochloride for 10 min before harvest. For harringtonine run-off, cells were treated with 2 µM harringtonine for the indicated time prior to incubating with 10 µg/ml puromycin dihydrochloride for 10 min. Total cell lysates were resolved by SDS-PAGE, transferred to the nitrocellulose membranes, and then incubated with a specific set of primary antibodies. For Phos-tag gel electrophoresis, protein samples were resolved by SDS-PAGE containing 10 µM Phos-tag (Wako) and 20 µM ZnCl_2_ (Sigma-Aldrich) according to the manufacturer’s instructions. Immunoreactive proteins were recognized by species-specific horseradish peroxidase-conjugated secondary antibodies (Jackson ImmunoResearch Laboratories, Thermo Fisher) and subsequently detected with Clarity Western ECL blotting substrate (Bio-Rad, Biomax) using iBright1500 (Thermo Fisher). Protein band intensities were measured from individual lanes using ImageJ software, subtracted by background signals, and then normalized to controls.

### Antibodies

Primary antibodies used in immunoblottings were anti-c-Abl (Santa Cruz Biotechnology, sc-56887), anti-actin (Santa Cruz Biotechnology, sc-47778), anti-AKT (Cell Signaling Technology, 4691), anti-p-AKT (Ser473, Cell Signaling Technology, 4060), anti-AMPK (Cell Signaling Technology, 2532), anti-p-AMPK (Thr172, Cell Signaling Technology, 2535), anti-EDF1 (Bethyl Laboratories, A304-039A; ABclonal, A2283), anti-EEF2 (Proteintech, 67550-1-Ig), anti-p-EEF2 (Thr56, Cell Signaling Technology, 2331), anti-EEF2K (Santa Cruz Biotechnology, sc-390710), anti-p-EEF2K (Ser366, Santa Cruz Biotechnology, sc-377536), anti-eIF2α (Santa Cruz Biotechnology, sc-133132), anti-p-eIF2α (Ser51; ABclonal, AP0745), anti-GAPDH (Proteintech, 10494-1-AP; Santa Cruz Biotechnology, sc-47724), anti-HA (Proteintech, 51064-2-AP), anti-JNK (Santa Cruz Biotechnology, sc-7345), anti-p-JNK (Thr183/Tyr185; Santa Cruz Biotechnology, sc-6254), anti-LTN1 (Invitrogen, PA5-42315), anti-NEMF (Proteintech, 11840-1-AP), anti-p38 (Santa Cruz Biotechnology, sc-7972), anti-p-p38 (Tyr182; Santa Cruz Biotechnology, sc-166182), anti-PI3K (Cell Signaling Technology, 4257), anti-p-PI3K (Tyr458, Tyr199, Cell Signaling Technology, 4228), anti-puromycin (Merck Millipore, MABE343), anti-RPL4 (Santa Cruz Biotechnology, sc-100838), anti-RPS2 (Bethyl Laboratories, A303-794A), anti-RPS6 (Santa Cruz Biotechnology, sc-74459), anti-RPS10 (Abcam, ab151550), anti-S6K (Cell Signaling Technology, 9202), anti-p-S6K (Thr389, Cell Signaling Technology, 9206), anti-mTOR (Cell Signaling Technology, 2972), anti-p-mTOR (Ser2448; Cell Signaling Technology, 2971), anti-tubulin (Proteintech, 10094-1-AP; Developmental Studies Hybridoma Bank, E7), anti-ZAK (Bethyl Laboratories, A301-993A; ABclonal, custom-generated; Proteintech, 28761-1-AP), and anti-ZNF598 (Bethyl Laboratories, A305-108A; ABclonal, custom-generated). Secondary antibodies used in immunoblottings are goat anti-Mouse IgG (H + L) Secondary Antibody, HRP (Thermo Fisher, 31430), goat anti-Rabbit IgG (H + L) Secondary Antibody, HRP (Thermo Fisher, 31460); Peroxidase AffiniPure™ Donkey Anti-Mouse IgG (H + L) (Jackson ImmunoResearch Laboratories, 715-035-150), and Peroxidase AffiniPure™ Donkey Anti-Rabbit IgG (H + L) (Jackson ImmunoResearch Laboratories, 711-035-152).

### Quantitative transcript analysis

Total RNAs were purified using TaKaRa MiniBEST Universal RNA Extraction Kit according to the manufacturer’s instructions. RNA samples were reverse-transcribed using M-MLV transcriptase (Promega) with random hexamers. Diluted cDNA samples were quantitatively analyzed by real-time PCR (QuantStudio 1, Thermo Fisher) using Prime Q-Mastermix (GeNetBio) and gene-specific primer pairs. Primer sequences used in transcript analysis were: 5’-TCC TAT TCT GGG GTC ACC GT-3’ (ZAK_F), 5’-AGG AGA CGA GCT TCT GGA CT-3’ (ZAK_R), 5’-AAC CTC GAC AAA TGG TCC TG-3’ (ZNF598_F), 5’-GTC TTC GTC CTT GAG CTT CG-3’ (ZNF598_R), 5’-TGG TCA CCA GGG CTG CTT-3’ (GAPDH_F), 5’-TTG ACG GTG CCA TGG AAT T-3’ (GAPDH_R).

### Sucrose density gradient fractionation

Cells on a 100-mm dish were collected by centrifugation at 2000 rpm for 3 min at room temperature and washed twice with PBS containing 100 μg/ml CHX. Cell pellets were lysed in sucrose density gradient buffer (15 mM Tris-Cl pH 7.5, 15 mM MgCl_2_, 200 mM NaCl, 100 μg/ml CHX) containing 1% Triton X-100 (v/v), 1 mM PMSF, and 1 mM DTT at 4 °C for 15 min. After clarification by centrifugation, soluble extracts were incubated with 500 U micrococcal nuclease (New England Biolabs) and 1 mM CaCl_2_ for 30 min at 25 °C. The digestion was terminated by adding 2 mM EGTA. The extracts were loaded onto 10–40% (w/v) sucrose density gradient solution and centrifuged at 38,000 rpm for 120 min using an SW41Ti rotor (Beckman Coulter). Gradient samples were fraction-collected into 1.5 ml tubes using Minipuls 3 (Gilson) and a tube piercer (Brandel). UV absorbance at 250 nm was monitored continuously with a UV detector (REACH Devices). Protein samples were prepared from individual fractions by methanol-chloroform precipitation, resolved by SDS-PAGE, and then analyzed by immunoblotting.

### Quantitative image analysis

Transfected cells were seeded on lysine-coated coverslips. After drug treatment, cells were fixed in PBS containing 3.7% formaldehyde at room temperature for 15 min. Fixed cells were washed twice with PBS and permeabilized with PBS containing 0.1% Triton X-100 (PBS-T) at 4 °C for 15 min. After two washes with PBS, nuclei were stained with Hoechst 33258 for 5 min. The coverlips were washed twice with PBS-T and mounted on imaging slides using the VECTASHIELD antifade mounting medium (Vector Laboratories). Confocal images were obtained using the FV1000 (Olympus) and AX (Nikon) microscopes. Random fields of interest in each slide were scanned with identical imaging settings. The p38-KTR reporter expression in each cell was quantified by measuring fluorescence intensity in the cytoplasm vs. nucleus using ImageJ software. The relative distribution of the fluorescence reporter was calculated by the ratio of cytoplasmic to nuclear intensities (C/N ratio).

### DEG analysis

Microarray data for gene expression analyses in a cohort of CML patients (chronic phase, CP  =  42 patients; accelerated phase, AP  =  15 patients; blast phase, BP  =  36 patients) were obtained from Gene Expression Omnibus (GSE4170) [58] and analyzed with GEO2R (https://www.ncbi.nlm.nih.gov/geo/geo2r/).

### Statistical analysis

Statistical analyses were performed using GraphPad Prism (version 10.3.1) and R (version 4.2.3). Normality and equal variance were determined by Shapiro-Wilk and Brown-Forsythe tests, respectively. For two-sample comparisons, (1) parametric datasets with equal variance were analyzed by unpaired *t* test; (2) nonparametric datasets were analyzed by Mann–Whitney test; (3) the significance of the correlation between two variables was determined by Spearman correlation. For multiple-sample comparisons, (1) datasets with a low number of samples (n < 8) were analyzed by ordinary ANOVA with Tukey’s multiple comparisons or Dunnett’s multiple comparisons regardless of their normality or equal variance; (2) nonparametric datasets with equal variance were analyzed by Kruskal-Wallis test with Dunn’s multiple comparisons test; (3) nonparametric datasets with unequal variance were analyzed by aligned ranks transformation (ART) ANOVA with Wilcoxon rank sum test. The statistical details of each panel (i.e., sample size, statistical method used, and *P* values) were all summarized in Supplementary Table S2 and indicated in figure legends accordingly. No statistical calculation was conducted to determine the sample size.

## Results

### ZAK upregulation correlates with CML progression to blast phase

**Fig. 1:**
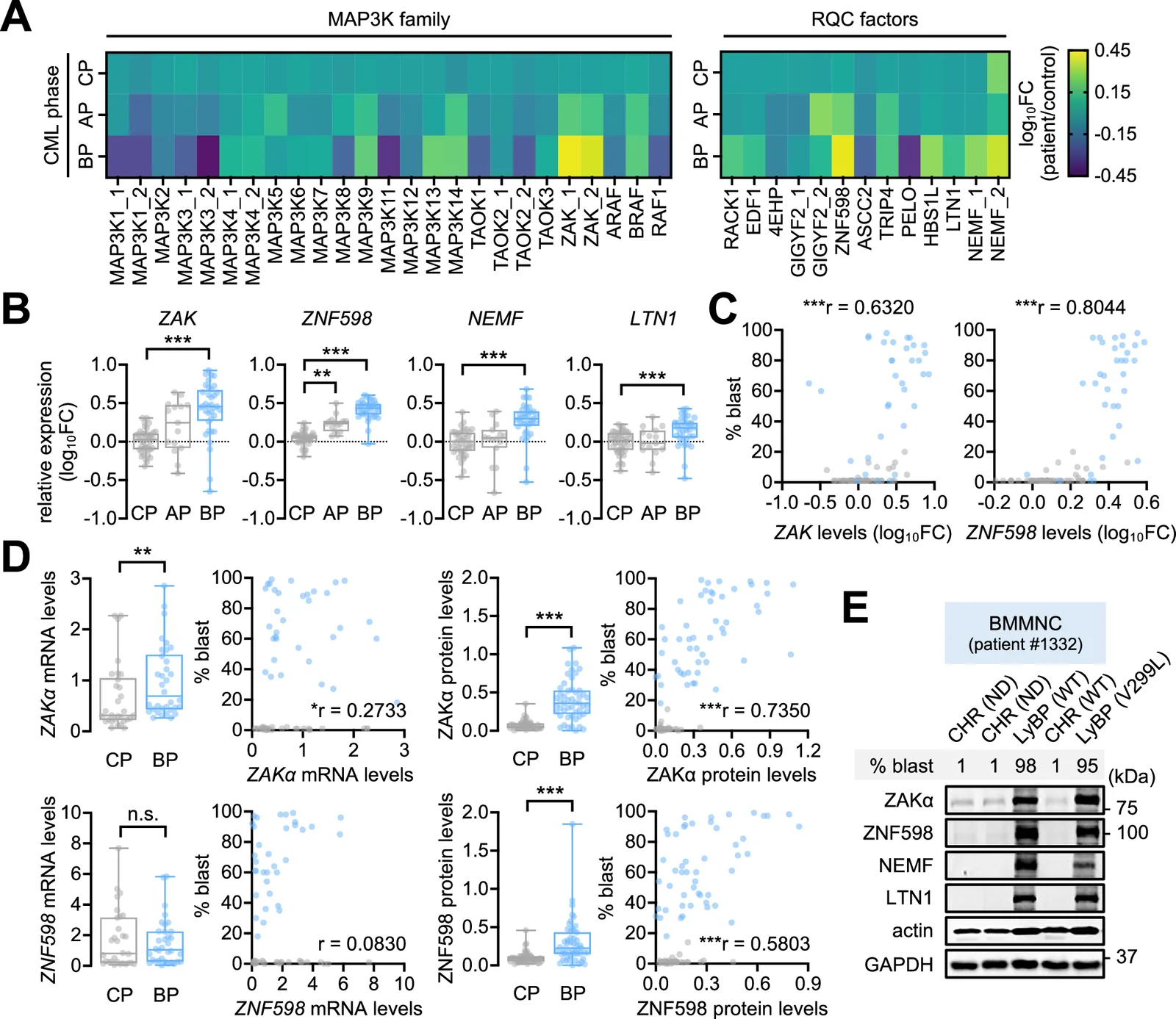
ZAK upregulation correlates with CML progression to blast phase. ZAK and a subset of RQC factors are selectively upregulated during CML progression. Microarray data for gene expression analyses in a cohort of CML patients (chronic phase, CP  =  42 patients; accelerated phase, AP  =  15 patients; blast phase, BP  =  36 patients) were obtained from Gene Expression Omnibus (GSE4170). Differential gene expression of MAP3K family members and RQC-relevant factors was visualized in heatmaps (**A**) and box plots (**B**). Relative expression levels in individual patients were calculated by normalizing to those in pooled CP samples and displayed as a log10 fold-change (log10FC). The box extends from the 25th to 75th percentiles with a median line, and the whiskers cover min to max values. **P < 0.01, ***P < 0.001 as determined by aligned ranks transformation (ART) 1-way ANOVA with Wilcoxon rank sum test (*ZAK*) or Kruskal-Wallis test with Dunn’s multiple comparisons test (*ZNF598*, *NEMF*, and *LTN1*). **C** The percentage of blast cells (% blast) positively correlates with *ZAK* or *ZNF598* mRNA levels in CML patients (*n* = 93; gray dots, CP or AP patients; blue dots, BP patients). ***P < 0.001 as determined by Spearman correlation. **D** BP patients display high ZAK expression at both mRNA and protein levels. ZAK expression was quantified in BMMNC from CML patients (*n* = 30 CP patients and 34 BP patie*n*ts for quantitative PCR analysis; *n* = 48 CP patients and 51 BP patie*n*ts for immunoblotting analysis). The abundance of each transcript was quantified using real-time PCR with gene-specific primer pairs and normalized to that of glyceraldehyde-3-phosphate dehydrogenase (*GAPDH*) in each patient sample. Relative *ZAK* mRNA levels were then calculated by normalizing to those in K562 cells (set as 1). Protein expression was quantified by the corresponding band intensity in immunoblotting (also see Supplementary Fig. S1). The relative abundance of ZAK protein was calculated by normalizing the ratio of ZAK to actin protein in each patient sample to that in K562 cells (set as 1). The percentages of blast cells positively correlate with ZAK expression at both mRNA and protein levels. n.s., not significant; **P < 0.01, ***P < 0.001 as determined by Mann–Whitney test (CP *vs*. BP) or Spearman correlation. **E** BP progression in each CML patient accompanies elevated expression of ZAKα protein and RQC factors. BMMNC samples were serially obtained from the patient #1332 at different CML stages. Protein expression was assessed by immunoblotting of total cell lysates (also see Supplementary Fig. S2). Specific BCR::ABL1 kinase domain mutations detected were given in parentheses. WT wild-type, ND not determined, CHR complete hematologic response, LyBP lymphoid blast phase.

A previous study profiled differentially expressed genes (DEGs) in a relatively large cohort of CML patients at distinct clinical stages [chronic phase (CP)  =  42 patients; accelerated phase (AP)  =  15 patients; blast phase (BP)  =  36 patients] [58]. Our analysis of the published dataset revealed that BP patients exhibited selective upregulation of *ZAK* and the rate-limiting RQC factor *ZNF598* [59–62] among other MAP3K family members or RQC-relevant genes (Fig. 1A, B). Moreover, the relative levels of *ZAK* or *ZNF598* mRNAs positively correlated with the abundance of immature blood cells (i.e., % blast cells) across individual CML patients (Fig. 1C). To validate these findings, we established an independent cohort of CML patients in South Korea (CP = 48 patients; BP = 51 patients) and performed gene expression analyses of patient-derived bone marrow mononuclear cells (BMMNCs). Immunoblotting of total cell lysates revealed that ZAK protein and a subset of RQC factors (i.e., ZNF598, NEMF, and LTN1) were readily detectable in BMMNCs from BP but not CP patients (Supplementary Fig. S1A, B). In particular, *ZAK* expression was significantly elevated in BP patients at both the transcript and protein levels and positively correlated with % blast cells (Fig. 1D), although we observed lineage-associated differences in relative abundance of *ZAK* mRNAs (i.e., myeloid vs. lymphoid) (Supplementary Fig. S1C). BP-specific upregulation of ZAK and RQC factor expression was similarly observed when BMMNC samples were serially collected from identical patients at different disease stages and analyzed by immunoblotting (Fig. 1E and Supplementary Fig. S2). These results strongly support the clinical relevance of ZAK expression in CML progression and suggest a possible role for ribosome collisions and downstream signaling pathways in CML pathogenesis.

### ZAK supports CML proliferation via AKT activation

**Fig. 2:**
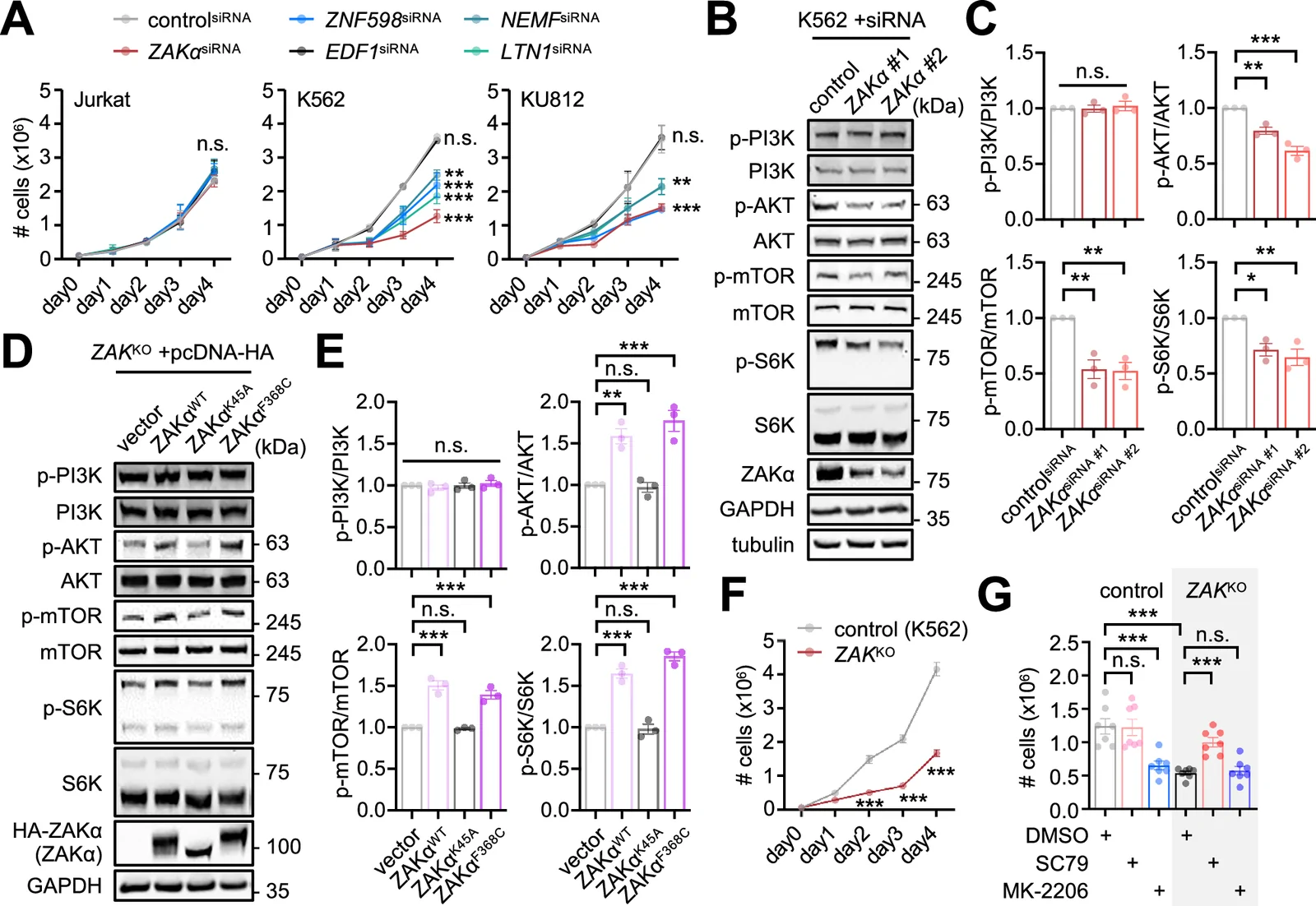
ZAK supports CML proliferation via AKT activation. **A** Depletion of ZAK or a subset of RQC factors reduces cell proliferation in CML cell lines (K562 and KU812) but not in the non-CML cell line (Jurkat). Cells were split into 60-mm culture dishes 48 h after siRNA transfection and daily counted. Data represent mean ± SEM (*n* = 3–6). n.s., not significant; **P < 0.01, ***P < 0.001 to control siRNA, as determined by 1-way ANOVA, Dunnett’s multiple comparisons test. **B**,** C** ZAK depletion attenuates AKT-mTOR signaling in K562 cells. Total cell lysates were prepared 48 h after siRNA transfection and immunoblotted with the indicated antibodies. The relative abundance of phosphorylated proteins was calculated by normalizing the ratio of phospho-specific to total signals to that in control siRNA (set as 1). Data represent mean ± SEM (*n* = 3). n.s., not sig*n*ificant; *P < 0.05, **P < 0.01, ***P < 0.001 as determined by 1-way ANOVA, Dunnett’s multiple comparisons test. **D, E** ZAK overexpression upregulates AKT-mTOR signaling in a ZAK kinase activity-dependent manner. *ZAK*-deleted K562 cells (*ZAK*^KO^) were transfected with expression vectors for wild-type (WT), kinase-dead (K45A), or constitutively active ZAK (F368C). Total cell lysates were prepared 48 h after transfection and immunoblotted with the indicated antibodies. The relative abundance of phosphorylated proteins was calculated by normalizing the ratio of phospho-specific to total signals to that in the vector control. Data represent mean ± SEM (*n* = 3). n.s., not significant; **P < 0.01, ***P < 0.001 as determined by 1-way ANOVA, Tukey’s multiple comparisons test. **F** *ZAK* deletion impairs cell proliferation in K562 cells. Control (K562) and *ZAK*^KO^ cells were split into 60-mm culture dishes and daily counted. Data represent mean ± SEM (*n* = 4). ***P < 0.001 to control on the same day, as determined by 2-way ANOVA, Tukey’s multiple comparisons test. **G** *ZAK* sustains K562 cell proliferation in an AKT activity-dependent manner. Control (K562) and *ZAK*^KO^ cells were counted 48 h after treatment with DMSO (vehicle control), SC79 (1 µM, AKT activator), or MK-2206 (1 µM, AKT inhibitor). Two-way ANOVA detected significant interaction effects of *ZAK* deletion with SC79 (*P* = 0.0156) or MK-2206 (*P* = 0.0003) on cell proliferation. Data represent mean ± SEM (*n* = 7). n.s., not significant; ***P < 0.001 as determined by Tukey’s multiple compariso*n*s test.

To investigate whether ZAK and RQC factors contribute to CML cell physiology, we employed BCR::ABL1-positive CML cell lines derived from BP patients (i.e., K562 and KU812). Immunoblotting analyses confirmed that both cell lines highly expressed ZAK proteins compared to non-CML cell lines (Supplementary Fig. S3A). K562 cells additionally displayed elevated levels of a subset of RQC factors (i.e., ZNF598, NEMF, and LTN1). We manipulated the expression of ZAK and RQC factors to examine their genetic effects on BCR::ABL1-dependent cell viability. Transient transfection with small interfering RNAs (siRNAs) effectively depleted ZAK or individual RQC factors in both CML and non-CML cells (Supplementary Fig. S3B; K562 and KU812 for CML; Jurkat for non-CML). Notably, their depletion impaired cell proliferation only in the CML cell lines (Fig. 2A). It is unlikely that nonspecific RNAi overload caused these siRNA phenotypes, as depletion of another ribosome-collision sensor EDF1 had no significant effect on CML proliferation (Fig. 2A).

Considering that (1) BCR::ABL1 promotes mTOR activity via the PI3K-AKT pathway for protein synthesis and cell proliferation [63–65] and (2) ZAK can directly phosphorylate AKT [66], we hypothesized that ZAK may promote mTOR activity and support CML proliferation via AKT regulation. ZAK-depleted K562 cells indeed displayed lower phosphorylation levels of AKT, mTOR, and downstream S6K than control cells, whereas the upstream PI3K phosphorylation was comparably detected between the two cell lines (Fig. 2B, C). The *ZAK* effects on AKT and downstream effectors were consistently observed in *ZAK*-deleted K562 cells (*ZAK*^KO^) (Supplementary Fig. S4). Conversely, overexpression of wild-type (ZAK^WT^) and constitutively active ZAK (ZAK^F368C^), but not kinase-dead mutant (ZAK^K45A^) [12, 19, 67], selectively elevated the phosphorylation levels of AKT, mTOR, and S6K in *ZAK*^KO^ cells, indicating these phenotypes depend on the ZAK kinase activity (Fig. 2D, E). Finally, treatment of K562 cells with the AKT inhibitor MK-2206 phenocopied proliferation defects in *ZAK*^KO^ cells, whereas treatment of *ZAK*^KO^ cells with the AKT activator SC79 rescued their impaired proliferation (Fig. 2F, G). Taken together, these series of data indicate that CML cell proliferation depends specifically on ZAK-dependent AKT activation, possibly explaining ZAK upregulation in BP patients.

### ZAK mediates CML apoptosis upon BCR::ABL1 inhibition

**Fig. 3:**
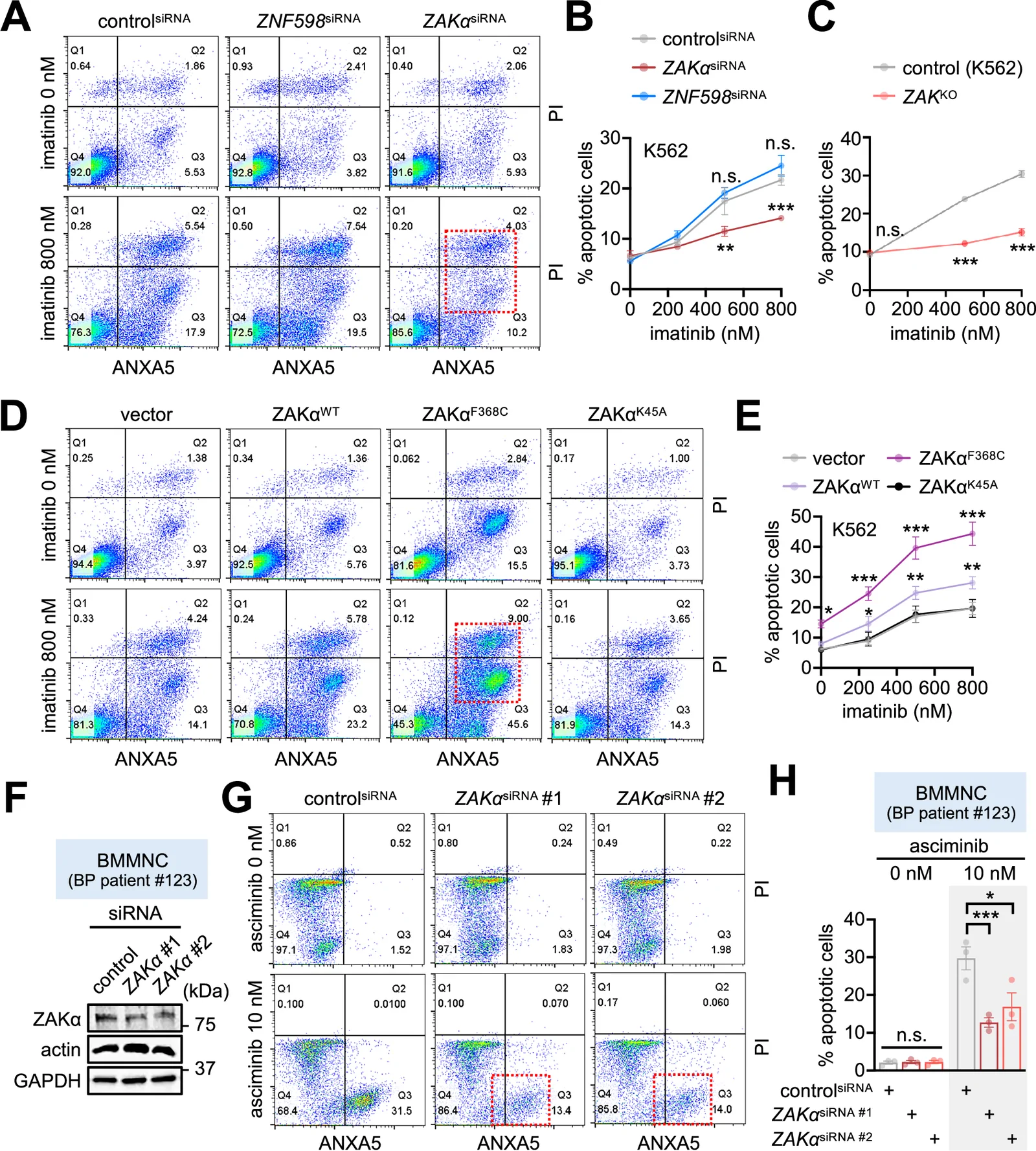
ZAK supports CML apoptosis upon BCR::ABL1 inhibition. **A**,** B** ZAK depletion suppresses imatinib-induced cell death in K562 cells. siRNA-transfected K562 cells were incubated with imatinib for 48 h before flow cytometry analysis. The percentage of apoptotic cells was scored by the intensity of FITC Annexin V (ANXA5) and propidium iodide (PI) stainings (*n* = 50,000 cells). Two-way ANOVA detected significant effects of *ZAK* (*P* = 0.0008) but not *ZNF598* (*P* = 0.2064) on % apoptotic cells. Data represent mean ± SEM (*n* = 3). n.s., not significant; **P < 0.01, ***P < 0.001 to control siRNA at given imatinib concentrations, as determined by Tukey’s multiple comparisons test. **C** *ZAK* deletion suppresses imatinib-induced cell death in K562 cells. Control (K562) and *ZAK*^KO^ cells were incubated with imatinib for 48 h before flow cytometry analysis. Two-way ANOVA detected significant effects of *ZAK* deletion on % apoptotic cells (*P* < 0.0001). Data represent mean ± SEM (*n* = 3). n.s., not significant; ***P < 0.001 to control at given imatinib concentrations, as determined by Tukey’s multiple comparisons test. **D**,** E** ZAK overexpression promotes imatinib-induced apoptosis in K562 cells. K562 cells were transfected with expression vectors for wild-type (WT) or mutant ZAK proteins (F368C, constitutively active; K45A, kinase-dead). At 48 h posttransfection, cells were treated with imatinib and further incubated for 48 h before flow cytometry analysis (*n* = 50,000 cells). Two-way ANOVA detected sig*n*ificant effects of ZAK WT (*P* = 0.0001) and ZAK F368C (*P* < 0.0001) but not ZAK K45A (*P* = 0.8637) on % apoptotic cells. Data represent mean ± SEM (*n* = 3–4). *P < 0.05, **P < 0.01, ***P < 0.001 to vector controls at given imatinib concentrations, as determined by Tukey’s multiple comparisons test. **F**–**H** ZAK depletion suppresses asciminib-induced apoptosis in CML patient cells. BP patient-derived BMMNCs were transfected with the indicated siRNAs. At 48 h posttransfection, cells were treated with asciminib and further incubated for 48 h before protein or flow cytometry analyses (*n* = 10,000 cells). Two-way ANOVA detected significant interaction effects between *ZAK* depletion and asciminib on % apoptotic cells (*P* = 0.0009 for siRNA #1; *P* = 0.0271 for siRNA #2). Data represent mean +/- SEM (*n* = 3). n.s., not significant; *P < 0.05, ***P < 0.001 as determined by Tukey’s multiple comparisons test.

Somewhat unexpectedly, we observed a significant difference between ZAK and the RQC factor ZNF598 in their depletion phenotypes when CML cells were treated with BCR::ABL1 inhibitors (i.e., imatinib, asciminib) to induce apoptosis [40, 41, 68]. ZNF598 depletion had minimal impact on CML apoptosis upon BCR::ABL1 inhibition (Fig. 3A, B). In contrast, ZAK depletion markedly reduced imatinib-induced apoptosis in K562 cells, as determined by flow cytometry analyses of Annexin V (ANXA5)-positive apoptotic cells (Fig. 3A, B) and immunoblotting of cleaved caspase-3 (Supplementary Fig. S5A). The specificity of *ZAK* effects was further confirmed by (1) *ZAK* deletion in K562 cells (Fig. 3C, *ZAK*^KO^); (2) the ZAK inhibitor sorafenib [69, 70] (Supplementary Fig. S5B); (3) independent *ZAK* siRNAs and a different CML cell line (i.e., KU812) (Supplementary Fig. S5C, D); and (4) a different class of the BCR::ABL1 allosteric inhibitor asciminib [68, 71] (Supplementary Fig. S5E, F). Overexpression of wild-type ZAK^WT^ or constitutively active ZAK^F368C^ enhanced imatinib-induced apoptosis in K562 cells (Fig. 3D, E). In fact, ZAK^F368C^ overexpression induced apoptosis even in the absence of BCR::ABL1 inhibition, suggesting that it may activate a pro-apoptotic pathway in CML cells. This pro-apoptotic effect required ZAK kinase activity, as (1) overexpression of kinase-dead ZAK^K45A^ did not potentiate imatinib-induced apoptosis (Fig. 3D, E); and (2) the K45A mutation masked pro-apoptotic activity of the F368C mutation in the ZAK^K45AF368C^ double mutant background regardless of imatinib treatment (Supplementary Fig. S5G, H).

Finally, we depleted ZAK expression in BP patient-derived BMMNCs and examined their sensitivity to BCR::ABL1 inhibition (Fig. 3F–H). Given that the patient displayed imatinib resistance, we treated the patient-derived BMMNCs with asciminib and quantified apoptotic cell death using flow cytometry. Of note, the patient-derived primary cultures showed two subpopulations with distinct propidium iodide (PI) staining (Fig. 3G; Supplementary Fig. S6A, groups #1 and #2), likely reflecting their intrinsic heterogeneity (e.g., cell size, granularity, membrane properties, and differentiation state). Nevertheless, asciminib treatment substantially reduced both populations while increasing the percentage of early apoptotic cells (Fig. 3G, H; Supplementary Fig. S6B). ZAK depletion significantly reduced asciminib-induced apoptosis in patient-derived BMMNCs (Fig. 3F–H), confirming our observations in ZAK-depleted CML cell lines. These findings define a ZAK-dependent pathway of CML apoptosis.

### BCR::ABL1 inhibition induces ZAK-dependent ribotoxic stress responses via mTOR silencing

**Fig. 4:**
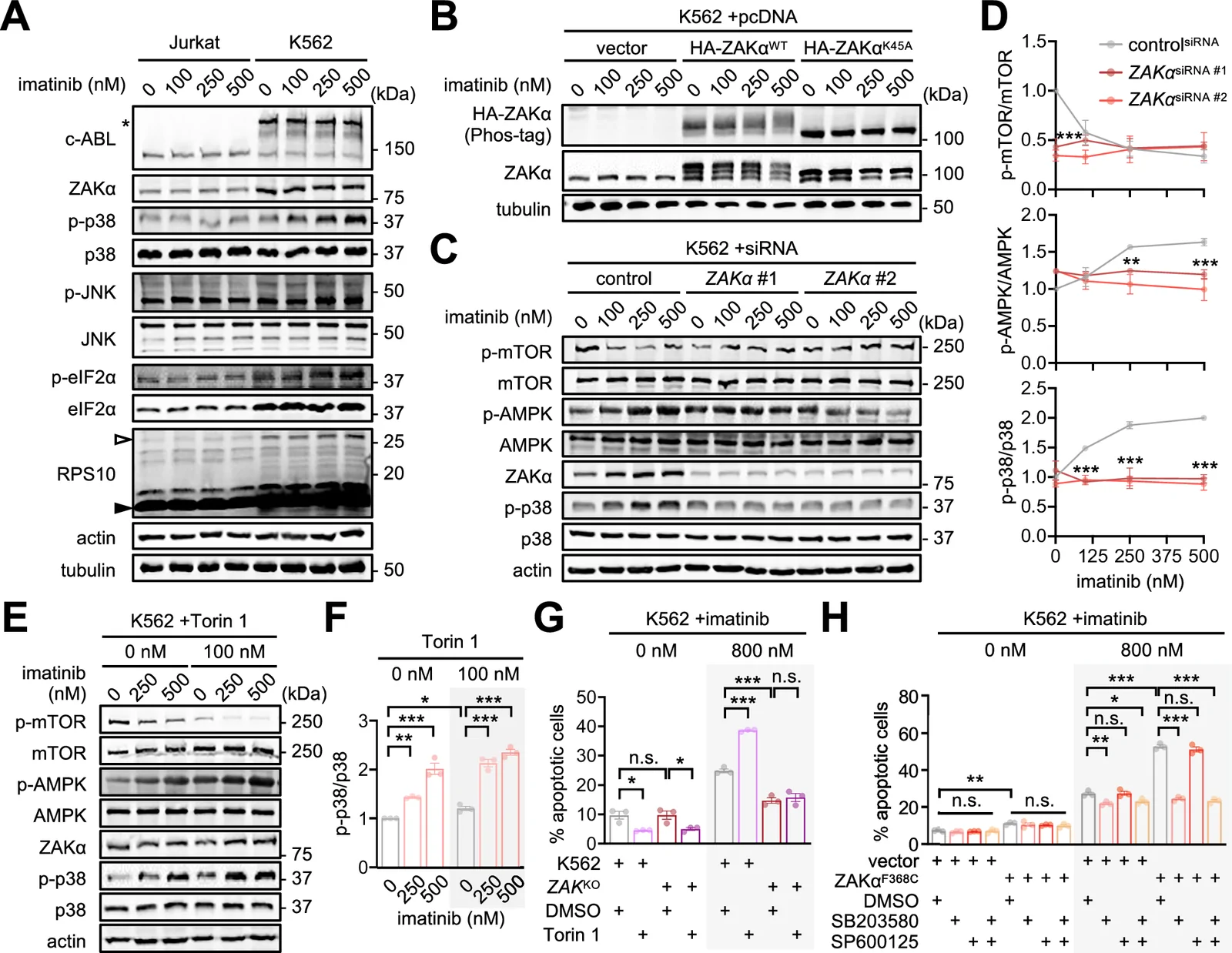
BCR::ABL1 inhibition induces ZAK-dependent ribotoxic stress responses via mTOR silencing. **A** Imatinib treatment promotes p38 phosphorylation and RPS10 ubiquitination in BCR::ABL1-positive K562 but not in BCR::ABL1-negative Jurkat cells. Jurkat and K562 cells were incubated with imatinib for 6 h before harvest. Total cell lysates were immunoblotted using the indicated antibodies. Asterisk, BCR::ABL1; white arrowhead, ubiquitinated RPS10; black arrowhead, RPS10. **B** Imatinib treatment induces phosphorylation of wild-type but not kinase-dead ZAKα proteins in K562 cells. K562 cells were transfected with HA-tagged expression vectors for wild-type (WT) or mutant ZAK proteins (K45A, kinase-dead). At 48 h posttransfection, cells were treated with imatinib and further incubated for 6 h before protein analysis. Where indicated, total cell lysates were resolved in the Phos-tag gel for phosphorylation-sensitive separation. **C**,** D** ZAK depletion abolishes imatinib-induced ribotoxic stress responses in K562 cells. siRNA-transfected K562 cells were incubated with imatinib for 6 h before protein analyses. Protein expression was quantified by the corresponding band intensity in immunoblotting. The relative abundance of phosphorylated proteins was calculated by normalizing the ratio of phospho-specific to total signals to that in control siRNA (0 nM imatinib; set as 1). Two-way ANOVA detected significant interaction effects between ZAK depletion and imatinib on mTOR (*P* = 0.0021 for siRNA #1; *P* = 0.0054 for siRNA #2), AMPK (*P* = 0.0001 for siRNA #1; *P* = 0.0009 for siRNA #2), and p38 phosphorylation (*P* < 0.0001 for both siRNAs). Data represent mean +/- SEM (*n* = 3). **P < 0.01, ***P < 0.001 to control siRNA at given imatinib concentrations, as determined by Tukey’s multiple comparisons test. **E**,** F** mTOR inhibition facilitates p38 phosphorylation in imatinib-treated K562 cells. K562 cells were incubated with the mTOR inhibitor Torin1 and imatinib for 6 h before harvest. p38 phosphorylation levels were quantified as above. Two-way ANOVA detected significant interaction effects between Torin1 and imatinib on p38 phosphorylation (*P* = 0.0059). Data represent mean ± SEM (*n* = 3). *P < 0.05, **P < 0.01, ***P < 0.001 as determined by Tukey’s multiple comparisons test. **G** *ZAK* deletion masks Torin1-induced apoptosis in imatinib-treated K562 cells. Control (K562) and *ZAK*^KO^ cells were incubated with Torin1 (100 nM) and imatinib for 48 h before flow cytometry analysis of apoptotic cells. Two-way ANOVA detected significant interaction effects between *ZAK* deletion and Torin1 on % apoptotic cells only in the presence of imatinib treatment (*P* = 0.0001). Data represent mean ± SEM (*n* = 3). n.s., not significant; *P < 0.05, ***P < 0.001 as determi*n*ed by Tukey’s multiple comparisons test. **H** p38 inhibition blunts ZAK-dependent apoptosis in imatinib-treated K562 cells. K562 cells were transfected with expression vector for constitutively active ZAK (F368C). At 48 h posttransfection, cells were treated with the indicated combinations of SB203580 (1 µM, p38 inhibitor), SP600125 (1 µM, JNK inhibitor), and imatinib for 48 h before flow cytometry analysis of apoptotic cells. Two-way ANOVA detected significant interaction effects of ZAK with SB203580 (*P* < 0.0001) or co-treatment of SB203580 and SP600125 (*P* < 0.0001) on % apoptotic cells only in the presence of imatinib treatment. Data represent mean ± SEM (*n* = 3). n.s. not significant; *P < 0.05, **P < 0.01, ***P < 0.001 as determined by Tukey’s multiple compariso*n*s test.

We hypothesized that BCR::ABL1 inhibition triggers ZAK-dependent RSR, leading to CML cell apoptosis. To test this idea, we examined the post-translational modifications of cellular factors involved in ribosome collision and ZAK-dependent RSR using immunoblotting with phosphorylation-specific antibodies or by electrophoretic mobility shifts (e.g., Phos-tag gels). Treatment of K562 cells with increasing amounts of imatinib elevated the relative levels of phosphorylated p38 and ubiquitinated RPS10 proteins (Fig. 4A), two molecular markers of ribosome collision via RSR and RQC activation, respectively [12, 13, 60–62]. BCR::ABL1 inhibition also increased phosphorylation of both endogenous and epitope-tagged ZAK proteins (i.e., ZAK activation) [12, 13, 19, 23, 27], as evidenced by their slower migration in Phos-tag gel electrophoresis (Fig. 4B and Supplementary Fig. S7A). The phosphorylation-relevant mobility shift of endogenous ZAK, however, displayed a bell-shaped dosage response to imatinib treatment, differing from the dose-dependent phosphorylation of epitope-tagged ZAK. In fact, the proteasome inhibitor MG132 elevated relative levels of slowly migrating endogenous ZAK in imatinib-treated cells (Supplementary Fig. S7A), consistent with the previous finding that hyperphosphorylated ZAK undergoes proteasomal degradation [11]. We reason that phosphorylation-coupled decay kinetics of endogenous ZAK may be distinct from those of overexpressed ZAK since a heterologous promoter drives high transgene expression compared to endogenous ZAK levels (Fig. 4B). We additionally confirmed that imatinib-induced ZAK phosphorylation was dependent on its kinase activity, as the kinase-dead mutant exhibited no mobility shifts comparable to that of the wild-type protein (Fig. 4B). Neither p38 phosphorylation nor RPS10 ubiquitination was observed when BCR::ABL1-negative Jurkat cells were treated with imatinib (Fig. 4A). Conversely, ectopic expression of the BCR::ABL1 transgene in Jurkat cells was sufficient to induce p38 phosphorylation and RPS10 ubiquitination upon imatinib treatment (Supplementary Fig. S7B, C).

The pathogenic BCR::ABL1 kinase activates multiple signaling pathways to drive leukemogenesis [32, 34, 35], including the PI3K-AKT-mTOR pathway [37–39]. In fact, the mTOR-AMPK pathway has been shown to constitute ZAK-dependent RSR when metabolic stress conditions (e.g., nutrient deprivation) induce ribosome stalling [26]. We therefore examined whether ZAK shapes phosphorylation signaling in response to BCR::ABL1 inhibition through these overlapping molecular pathways. Imatinib treatment of K562 cells decreased the relative phosphorylation levels of mTOR (p-mTOR) while increasing phosphorylation of both p38 and AMPK (p-p38 and p-AMPK) in a dose-dependent manner (Fig. 4C, D). ZAK depletion markedly reduced the basal levels of p-mTOR and blunted all phosphorylation responses to imatinib. These ZAK-dependent effects were consistently observed when BCR::ABL1 activity was inhibited by imatinib in (1) *ZAK*-deleted K562 cells (Supplementary Fig. S8A, B), (2) another CML cell line KU812 (Supplementary Fig. S8C), and (3) BCR::ABL1-overexpressing Jurkat cells (Supplementary Fig. S8D, E); or (4) by asciminib in K562 cells (Supplementary Fig. S8F). Moreover, *ZAK* was necessary for imatinib-induced p38 phosphorylation, as independently confirmed by confocal imaging of a p38 kinase translocation reporter that quantified p38 activation at the single-cell level [56, 72] (Supplementary Fig. S8G).

While BCR::ABL1 inhibition induces p38 phosphorylation in CML cell lines, p38 inhibition suppresses anti-leukemic responses [73, 74]. The mTOR inhibitor rapamycin has also been shown to enhance imatinib-induced apoptosis in CML [39, 45–47, 74–76]. Under our experimental conditions, the mTOR inhibitor Torin 1 similarly facilitated imatinib-dependent p38 activation (Fig. 4E, F) and CML apoptosis (Fig. 4G). Importantly, *ZAK* deletion silenced the effects of mTOR inhibition on the imatinib-induced cell death (Fig. 4G), although we do not rule out the possibility of ZAK-independent mechanisms responsible for apoptosis in *ZAK*^KO^ cells. We further found that the p38 inhibitor SB203580, but not the JNK inhibitor SP600125, suppressed imatinib-induced apoptosis in CML, particularly when the hyperactive ZAK mutant exaggerated the effects of BCR::ABL1 inhibition (Fig. 4H). The p38-selective effects were consistent with barely detectable induction of JNK phosphorylation upon BCR::ABL1 inhibition (Fig. 4A), as observed in previous studies [24, 73]. By contrast, JNK has been shown to mediate ZAK-dependent apoptosis during UV-induced RSR [11, 22]. Considering that anisomycin treatment induces both SAPK phosphorylation in K562 cells (Supplementary Fig. S9), it is plausible to reason that RSR-triggering cues (i.e., type, intensity, duration) and their downstream effects on translation flux can shape the kinetics and amplitude of the two SAPK activation differentially and determine subsequent effector pathways accordingly (e.g., cell cycle arrest, inflammation, apoptosis) [21, 69]. The fact that p38 antagonizes JNK may also generate cell type- and cue-specific heterogeneity in SAPK activation during RSR [77]. Together, these findings reveal a crucial role for ZAK in coordinating mTOR-relevant cellular responses to BCR::ABL1 inhibition, thereby promoting CML apoptosis via p38 activation.

### BCR::ABL1 inhibition induces EEF2 phosphorylation and ribosome collisions

**Fig. 5:**
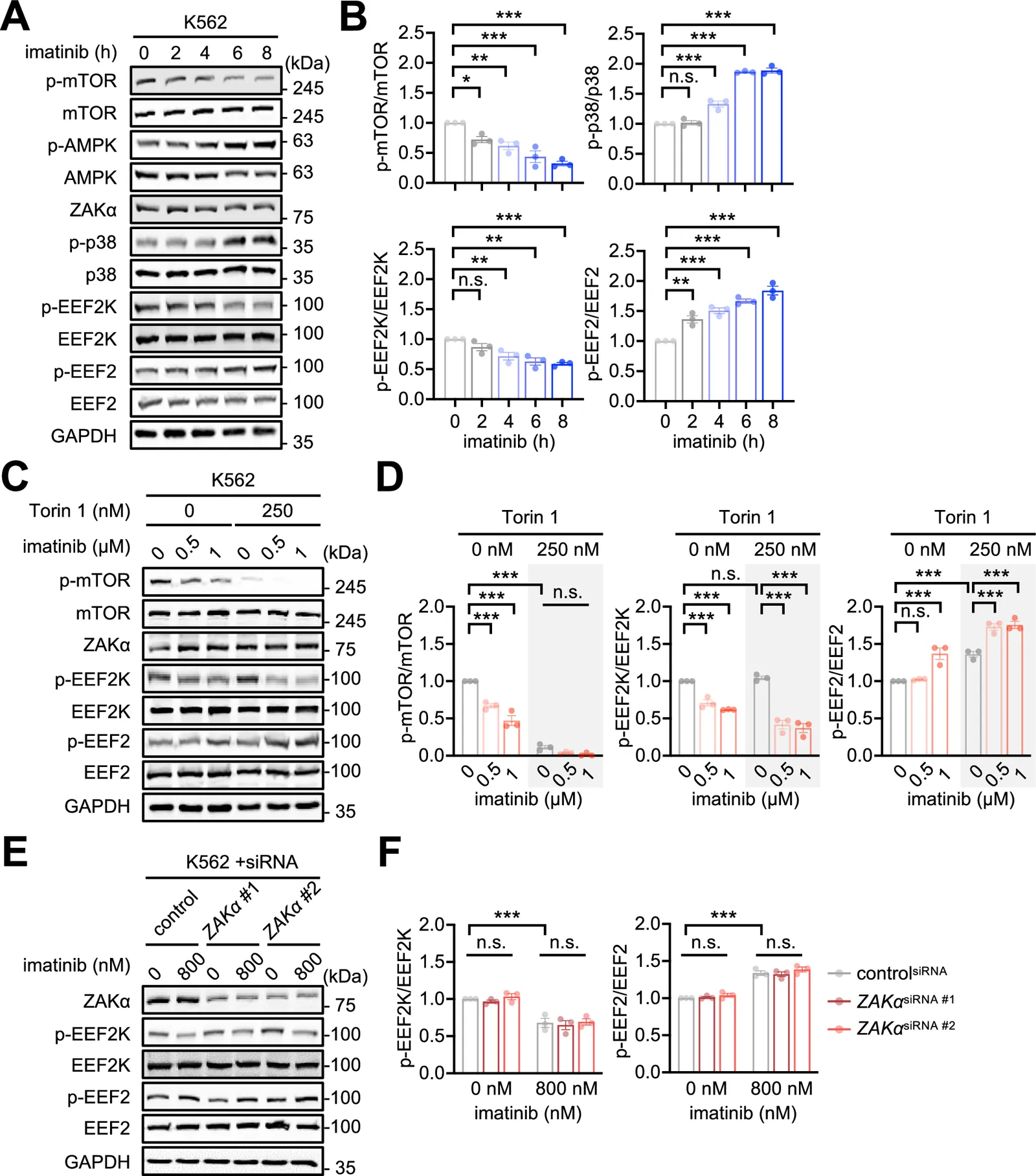
BCR::ABL1 inhibition induces EEF2 phosphorylation via mTOR silencing. **A**,** B** Imatinib treatment of K562 cells reduces the phosphorylation levels of mTOR and EEF2K proteins while elevating those of p38 and EEF2 proteins. K562 cells were incubated with imatinib (1 µM) for the indicated time before harvest. Phosphorylation levels of mTOR, EEF2K, EEF2, and p38 proteins were quantified as in Fig. 4. Data represent mean ± SEM (*n* = 3). n.s., not significant; *P < 0.05, **P < 0.01, ***P < 0.001 as determined by 1-way ANOVA, Dunnett’s multiple comparisons test. **C**,** D** mTOR inhibition exaggerates imatinib-induced changes in EEF2K-EEF2 signaling. K562 cells were incubated with Torin 1 and imatinib for 8 h before harvest. Two-way ANOVA detected significant interaction effects between Torin 1 and imatinib on mTOR (*P* < 0.0001), EEF2K (*P* = 0.0021), and EEF2 phosphorylation (*P* = 0.0034). Data represent mean ± SEM (*n* = 3). n.s., not significant; ***P < 0.001 as determined by Tukey’s multiple comparisons test. **E**,** F** ZAK depletion had minimal effects on imatinib-induced changes in EEF2K-EEF2 signaling. siRNA-transfected K562 cells were incubated with imatinib for 8 h before protein analyses. Two-way ANOVA detected no significant interaction effects between ZAK and imatinib on EEF2K and EEF2 phosphorylation. Data represent mean ± SEM (*n* = 3). n.s., not significant; ***P < 0.001 as determi*n*ed by Tukey’s multiple comparisons test.

How can mTOR silencing trigger ZAK-dependent RSR upon BCR::ABL1 inhibition? Eukaryotic elongation factor 2 kinase (EEF2K) is a critical regulator of protein translation and cellular stress responses [78, 79]. EEF2K suppresses translation elongation by phosphorylating eukaryotic elongation factor 2 (EEF2) at Thr56 [80, 81]. Notably, molecular pathways downstream of mTOR activation include ribosomal protein S6 kinase, which directly phosphorylates EEF2K at Ser366 to inhibit its activity [82]. We thus hypothesized that BCR::ABL1 inhibition and consequent mTOR silencing may slow translation elongation via the EEF2K-EEF2 axis, thereby increasing the likelihood of ribosome collisions and ZAK-dependent RSR activation. Imatinib treatment of K562 cells indeed reduced the relative levels of phosphorylated EEF2K (p-EEF2K) at Ser366 while increasing phosphorylated EEF2 (p-EEF2) (Fig. 5A, B). The mTOR inhibitor Torin 1 alone had negligible effects on p-EEF2K levels at the concentration tested (Fig. 5C, D). Nonetheless, Torin 1 modestly elevated p-EEF2 levels, suggesting that mTOR inhibition may promote EEF2K activity through additional mechanisms (e.g., mTOR-dependent EEF2K phosphorylation at Ser78 or Ser396) [82]. Co-treatment of K562 cells with Torin 1 and imatinib synergistically lowered p-EEF2K levels at Ser366 while further elevating p-EEF2 levels (Fig. 5C, D). These observations were consistent with Torin 1 effects on imatinib-induced p38 phosphorylation (Fig. 4E, F), supporting our model that EEF2 phosphorylation acts downstream of the BCR::ABL1-mTOR pathway. Although ZAK promotes mTOR activity via AKT activation (Fig. 2B–E; Supplementary Fig. S4), the *ZAK* effects per se may not be strong enough to detectably modulate downstream p-EEF2K or p-EEF2 levels (Fig. 5E, F), placing the EEF2K-EEF2 axis upstream of ZAK in this pathway.

**Fig. 6:**
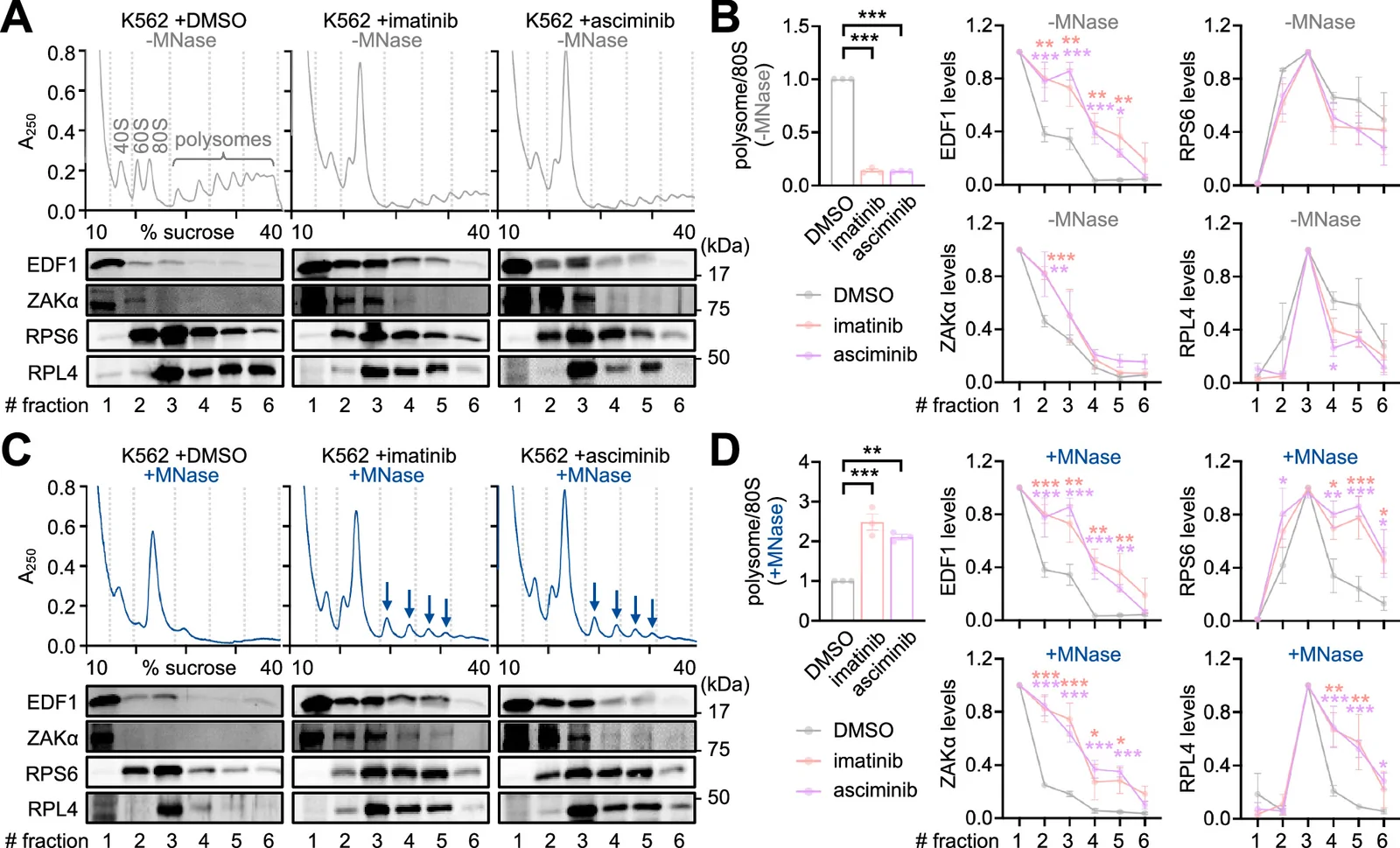
BCR::ABL1 inhibition induces ribosome collisions. **A**,** B** BCR::ABL1 inhibition reduces the ratio of polysome to monosome (80S) levels while translocating the ribosome collision sensor proteins (i.e., EDF1 and ZAK) to ribosomal fractions in the sucrose-density gradient profile of ribosomal populations. K562 cells were treated with BCR::ABL1 inhibitors (1 μM imatinib or 20 nM asciminib) or DMSO (vehicle control) for 6 h before harvest. Soluble cell lysates were loaded onto a 10–40% sucrose density gradient for biochemical fractionation by ultracentrifugation. Absorbance at 250 nm was continuously monitored during fraction collection (**A**, top). Total areas for polysomal peaks were quantified from the absorbance profile and normalized to that for the 80S peak (**B**, left top). Data represent mean ± SEM (*n* = 3). **P < 0.01, ***P < 0.001 as determined by 1-way ANOVA, Dunnett’s multiple comparisons test. Protein samples were precipitated from each fraction (dotted lines) and analyzed by immunoblotting (A, bottom). Relative protein levels in individual fractions were calculated by normalizing to the peak fraction per condition (set as 1). Data represent mean ± SEM (*n* = 3). *P < 0.05, **P < 0.01, ***P < 0.001 to the vehicle control in each fraction, as determined by 2-way ANOVA, Tukey’s multiple comparisons test. **C**,** D** BCR::ABL1 inhibition generates nuclease-resistant ribosomal fractions in the sucrose-density gradient profile of ribosomal populations. Soluble cell lysates from drug-treated K562 cells were incubated with micrococcal nuclease (MNase, 500 U) for 30 min to digest polysomal mRNAs into ribosome-protected ones before loading onto a 10–40% sucrose density gradient. Absorbance profiles and relative protein levels along a sucrose-density gradient were quantitatively analyzed as above. Blue arrows, MNase-resistant polysomes.

High p-EEF2 levels in BCR::ABL1-inhibited K562 cells were actually associated with low translation flux compared to control cells, as measured by puromycin labeling of nascent polypeptides (Supplementary Fig. S10A). Translation in BCR::ABL1-negative Jurkat cells was insensitive to BCR::ABL1 inhibitors, confirming the specific effects of BCR::ABL1 inhibition on global protein synthesis. Harringtonine run-off of the translating ribosomes [83] further validated that imatinib-treated K562 cells exhibited slow translation elongation compared to vehicle-treated control cells (Supplementary Fig. S10B), functionally coupling the modest increase in p-EEF2 levels to low elongation rate in BCR::ABL1-inhibited cells. We biochemically fractionated cell lysates using a sucrose-density gradient and compared the relative distribution of ribosomal populations (i.e., monosomes vs. actively translating polysomes) under distinct experimental conditions. Treatment of K562 cells with BCR::ABL1 inhibitors led to a decrease in polysome levels and a corresponding elevation of 80S monosome levels (Fig. 6A, B), consistent with a previous observation [76]. The reduced polysome-to-monosome ratio reflected impaired protein synthesis arising from mTOR effects on translation inhibition (i.e., reduced ribosome loading at the initiation step following BCR::ABL1 inhibition) [39, 84, 85]. Micrococcal nuclease (MNase) digestion of control cell lysates shifted the majority of polysomes to the 80S monosome fraction (Fig. 6C), suggesting that control polysomes were actively translating and therefore readily accessible to MNase [21]. In contrast, polysomes from BCR::ABL1-inhibited cells showed resistance to MNase treatment with a limited shift to the monosomal fraction compared to vehicle-treated controls (Fig. 6C, D; blue arrows).

Notably, BCR::ABL1 inhibition also caused EDF1 and ZAK to shift into ribosomal fractions in the standard sucrose-gradient profiles (Fig. 6A, B), indirectly indicating ribosome collisions [11, 13, 86, 87]. The nuclease-resistant ribosomal populations (i.e., disomes and polysomes) indeed retained these collision sensor proteins following BCR::ABL1 inhibition (Fig. 6C, D). Of note, it has been shown that the steady-state distribution of ZAK across sucrose gradients varies depending on cellular context (e.g., cell line, expression levels, and kinase activity) [11, 13, 15, 88]. We reason that these discrepancies can arise from differences in baseline ZAK activity since ZAK biochemically engages collided ribosomes and once activated, hyperphosphorylated ZAK is eventually released from ribosomes [11–13, 88]. Our transgene analysis further confirmed that (1) under baseline conditions, the kinase-dead mutant ZAK^K45A^ is more readily detected in polysomal fractions of K562 cell lysates than ZAK^WT^ and (2) only the transgenic ZAK^WT^ displays an imatinib-sensitive ribosome association comparable to endogenous ZAK (Supplementary Fig. S11). Collectively, these pieces of our evidence strongly support a model that BCR::ABL1 inhibitors exhibit dual effects on translation initiation and elongation to decrease general translation flux, and the mTOR-EEF2K-EEF2 pathway downstream of BCR::ABL1 inhibition is responsible for slow translation elongation, ribosome collisions, and ZAK-dependent RSR activation to promote CML apoptosis.

### Pharmacological manipulations of ribosome collisions tune CML sensitivity to BCR::ABL1 inhibition

**Fig. 7:**
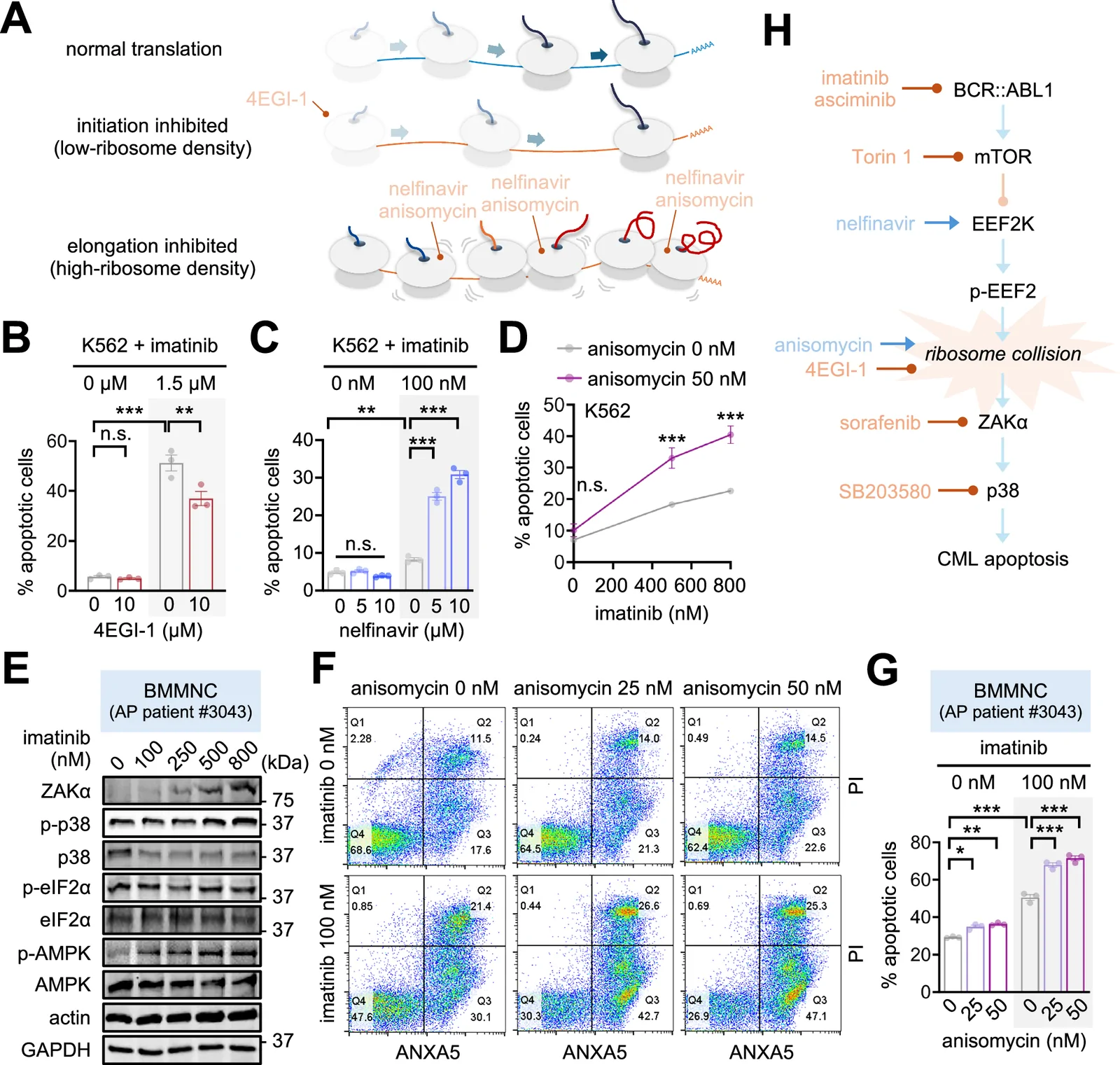
Pharmacological manipulations of ribosome collisions tune CML sensitivity to BCR::ABL1 inhibition. **A** A model for pharmacological manipulations of translating-ribosome density and ribosome collisions by distinct translation inhibitors. **B** Inhibition of cap-dependent translation initiation reduces imatinib-induced CML apoptosis. K562 cells were treated with the eIF4E-eIF4G inhibitor 4EGI-1 and imatinib for 48 h before flow cytometry analysis of apoptotic cells using FITC ANXA5 and PI stainings. Two-way ANOVA detected significant interaction effects between 4EGI-1 and imatinib on % apoptotic cells (*P* = 0.0132). Data represent mean ± SEM (*n* = 3). n.s., not significant; **P < 0.01, ***P < 0.001 as determined by Tukey’s multiple comparisons test. **C** EEF2K activation promotes imatinib-induced apoptosis in K562 cells. K562 cells were incubated with the EEF2K activator nelfinavir and imatinib for 48 h before flow cytometry analysis of apoptotic cells. Two-way ANOVA detected significant interaction effects between nelfinavir and imatinib on % apoptotic cells (*P* < 0.0001). Data represent mean ± SEM (*n* = 3). n.s., not significant; **P < 0.01, ***P < 0.001 as determined by Tukey’s multiple comparisons test. **D** Reversible inhibition of translation elongation exacerbates apoptosis in imatinib-treated K562 cells. K562 cells were incubated with the translation elongation inhibitor anisomycin and imatinib for 48 h before flow cytometry analysis of apoptotic cells. Two-way ANOVA detected significant interaction effects between anisomycin and imatinib on % apoptotic cells (*P* = 0.0072). Data represent mean +/- SEM (*n* = 3). n.s., not significant; ***P < 0.001 to vehicle control (0 nM anisomycin) at given imatinib concentrations, as determined by Tukey’s multiple comparisons test. **E** BCR::ABL1 inhibition induces ZAK-relevant RSR in CML patient cells. AP patient-derived BMMNCs were treated with imatinib for 6 h before immunoblotting analyses with the indicated antibodies. **F, G** Co-treatment of the translation elongation inhibitor anisomycin and imatinib synergistically promotes apoptosis in CML patient cells. AP patient-derived BMMNCs were incubated with anisomycin and imatinib for 48 h before flow cytometry analysis of apoptotic cells. Two-way ANOVA detected significant interaction effects between anisomycin and imatinib on % apoptotic cells (*P* = 0.0005 for 25 nM anisomycin; *P* = 0.0117 for 50 nM anisomycin). Data represent mean ± SEM (*n* = 3). *P < 0.05, **P < 0.01, ***P < 0.001 as determined by Tukey’s multiple comparisons test. **H** A working model for the BCR::ABL1 pathway to ribosome collision, ZAK-dependent RSR, and CML apoptosis. Blue, activating signals or activators; orange, inhibitory signals or inhibitors.

Given the model above, we predicted that partial inhibition of translation elongation would promote ribosome pausing and collisions, thereby enhancing the anti-leukemic effects of BCR::ABL1 inhibitors via the ZAK-dependent RSR (Fig. 7A). Conversely, partial inhibition of translation initiation may reduce the density of translating ribosomes on individual mRNAs, thereby limiting the molecular substrate available for ZAK-dependent RSR activation (Fig. 7A). To test this hypothesis, we employed distinct classes of translation inhibitors. The EIF4E-EIF4G interaction inhibitor 4EGI-1 selectively blocks cap-dependent translation initiation [89]. As expected, 4EGI-1 treatment suppressed imatinib-induced apoptosis in K562 cells (Fig. 7B). In contrast, co-treatment of imatinib with either the EEF2K activator nelfinavir [90] (Fig. 7C) or the translation elongation inhibitor anisomycin (Fig. 7D) synergistically enhanced apoptosis in K562 cells. These effects were unlikely to result from non-specific cytotoxicity of general translation inhibitors (i.e., 4EGI-1, nelfinavir, anisomycin), as low-dose treatment with these compounds alone barely induced K562 apoptosis under our experimental conditions (Fig. 7B–D).

HHT is a natural alkaloid approved by the FDA for CML treatment, particularly in patients resistant to BCR::ABL1 inhibitors [52, 53, 91]. HHT stalls the ribosome at the initiation step by interfering with ribosomal translocation [92]. Given that ribosome stalling is sufficient to activate ZAK and induce SAPK phosphorylation (i.e., JNK and p38) [12, 26, 27], we hypothesized that HHT treatment may also induce ZAK-dependent RSR to kill CML cells. HHT treatment of K562 cells indeed promoted SAPK phosphorylation in a dose-dependent manner, while ZAK depletion markedly suppressed HHT-induced SAPK phosphorylation and K562 apoptosis (Supplementary Fig. S12A, B). Polysome profiling further revealed that HHT treatment potently shifted polysome peaks toward to the 80S monosome (Supplementary Fig. S12C), consistent with ribosome pausing during translation initiation. However, HHT-treated K562 cells did not display any molecular signatures associated with ribosome collisions (e.g., MNase-resistant ribosomes; polysomal shifts of the collision sensors EDF1 and ZAK) comparable to BCR::ABL1-inhibited cells (Supplementary Fig. S12C, D). These results indicate that HHT and BCR::ABL1 inhibitors have distinct mechanisms of action in ZAK-mediated CML apoptosis. To further determine whether ZAK-dependent RSR and its pharmacological manipulation work in the primary CML patient cells, we employed AP patient-derived BMMNCs. Imatinib treatment of AP patient cells increased the relative expression levels of ZAK, p-p38, and p-AMPK, suggesting ZAK-mediated RSR activation (Fig. 7E). Moreover, co-treatment with the translation elongation inhibitor anisomycin substantially potentiated imatinib-induced apoptosis in patient-derived BMMNCs (Fig. 7F, G). Together, our findings demonstrate that the cytotoxic effects of BCR::ABL1 inhibitors can be modulated by pharmacologically manipulating the translating-ribosome density or the likelihood of ribosome pausing, supporting the clinical relevance of our working model (Fig. 7H).

## Discussion

Our study uncovers a translation-dependent stress mechanism that substantially shapes the therapeutic response of CML to BCR::ABL1 inhibition. More specifically, we demonstrate that pharmacological inhibition of BCR::ABL1 induces ribosome collisions, activates ZAK-dependent RSR, and triggers downstream stress-activated p38 signaling, ultimately promoting CML apoptosis. These findings establish ribosome collision-induced stress signaling as a key effector pathway engaged by BCR::ABL1 inhibitors in CML treatment. Moreover, we show that modulating the kinetic state of translating ribosomes, rather than overall protein synthesis, may serve as an anti-leukemic strategy distinct from conventional translation inhibitors with potential applicability to a broader spectrum of malignancies.

Clinical analyses of two independent CML cohorts revealed that ZAK expression was markedly elevated in BP patients, correlating with disease severity and the abundance of immature blast cells. In fact, ZAK expression is relatively low in hematopoietic stem cells but modestly increases along the myeloid differentiation trajectory of normal hematopoiesis, particularly in granulocyte-monocyte progenitors (GMPs), monocytes, and myeloid dendritic cells [93–95]. Given that BP cells most closely resemble GMP-like progenitors [96], BP-specific ZAK elevation may reflect lineage-associated genetic programming. Our data show that both myeloid and lymphoid BP cells exhibit elevated ZAK levels, suggesting that beyond lineage-specific regulation, co-translational surveillance factors (e.g., ZAK, ZNF598, NEMF, and LTN1) are likely upregulated in response to the translational burden characteristic of highly proliferative blast cells. We thus speculate that such contexts may prime BP cells to readily activate ribosome collision-induced stress responses upon BCR::ABL1 inhibition, thereby increasing their apoptotic sensitivity.

Previous studies have implicated mTOR signaling and p38 activation in regulating the fate of CML cells [39, 76]. Activation of the PI3K-AKT-mTOR pathway downstream of BCR::ABL1 has been shown to promote protein synthesis, cell growth, and CML proliferation, whereas inhibition of mTOR signaling potentiates the cytotoxic effects of BCR::ABL1 TKIs [39, 45–47, 74–76]. On the other hand, p38 activation has been reported to mediate apoptosis following BCR::ABL1 inhibition [73, 74, 97]. However, the mechanistic link between translational control and stress kinase activation has remained unclear. Our findings establish a mechanistic model where BCR::ABL1 inhibition suppresses mTOR activity, promotes EEF2 phosphorylation via EEF2K, slows down translation, and subsequently induces ribosome collisions. The identification of ribosome collision as a pivotal intermediary event links upstream translational suppression with downstream ZAK-dependent RSR, thereby revealing a mechanistically coherent axis through which BCR::ABL1 TKIs activate apoptotic signaling and promote CML cell death.

Notably, we found that loss of *ZAK* function reduced mTOR phosphorylation in the absence of BCR::ABL1 inhibition. Hinted from a previous observation that ZAK directly phosphorylates the upstream AKT [66], we provide compelling evidence that ZAK supports CML proliferation by activating the AKT-mTOR pathway. The *ZAK* effects elucidate why CML progression accompanies ZAK upregulation. Yet our observations contrast with the previous finding that acute metabolic stress activates ZAK to inhibit mTOR activity via AMPK activation [26]. We speculate that distinct physiological states (i.e., highly proliferative oncogenic signaling environment vs. nutrient stress) may explain the opposing effects of ZAK on mTOR regulation. More specifically, BCR::ABL1 signaling constitutively activates the PI3K-AKT-mTOR pathway with minimal AMPK activation, whereas AMPK overrides mTOR signaling upon nutrient stress. We thus propose that ZAK exhibits diverse cellular functions in a context-dependent manner to coordinate translational stress and metabolic signaling, expanding the known repertoire of ZAK-regulated physiological processes [16, 98].

Our results demonstrate that not all forms of translation inhibition elicit identical cellular responses during CML treatment. Enhancing ribosome collisions through partial elongation inhibition sensitized CML cells to BCR::ABL1 inhibition, whereas suppressing translation initiation diminished apoptotic responses. These findings highlight that translation elongation slowdown, coupled with sustained ribosome loading, amplifies RSR signaling downstream of BCR::ABL1 inhibition. This distinction suggests that the kinetic characteristics of translation, rather than overall translational output, critically determine whether translational stress promotes adaptive survival or apoptotic commitment. Within this mechanistic framework, we provide a rationale for the clinical efficacy of the elongation inhibitor HHT in CML [52, 53]. Specifically, we showed that HHT treatment alone activated ZAK-dependent stress responses and induced CML apoptosis, supporting the notion that the translational stress pathway contributes to its therapeutic effect (albeit through a mechanism distinct from collision-inducing BCR::ABL1 inhibitors). Furthermore, the observed synergy between BCR::ABL1 TKIs and translation elongation inhibitors (e.g., anisomycin, nelfinavir) suggests that co-targeting ribosome dynamics could enhance anti-leukemic efficacy, particularly in refractory or resistant CML patients (i.e., those resistant to BCR::ABL1 TKIs).

Oncogene-driven cancers are characterized by elevated translation rates and increased susceptibility to translational perturbations [28–31]. It is therefore plausible that targeted inhibition of other oncogenic signaling (e.g., EGFR or ALK inhibitors in lung cancer; FLT3 inhibitors in acute myeloid leukemia) may similarly influence translating ribosome dynamics [99–101]. Future studies should thus investigate whether ribosome collision-induced stress responses contribute to therapeutic efficacy across a broader spectrum of malignancies. Strategies that exploit the vulnerability of translational flux in cancer cells may enhance treatment efficacy and help overcome drug resistance.

## Supplementary information

Supplementary Figures S1-S12

Supplementary Table S1

Supplementary Table S2

Supplementary Table S3

## Data Availability

All the numeric data generated in this study were included in Supplementary Table S3. Raw data files for flow cytometry analyses were deposited to Zenodo (https://doi.org/10.5281/zenodo.15448140).
