# Correction: BCR::ABL1 tyrosine kinase inhibitors induce ribosome collisions to activate ZAK-dependent ribotoxic stress and apoptosis in chronic myeloid leukemia

**DOI:** 10.1038/s41375-026-03166-z

**Published:** 2026-10-09

**Authors:** Jumin Park, Soo-Hyun Kim, Jongmin Park, Heeju Park, Hongtae Kim, Dong-Wook Kim, Chunghun Lim

**Affiliations:** 1 Department of Biological Sciences, Korea Advanced Institute of Science and Technology, Daejeon, Republic of Korea; 2 Leukemia Omics Research Institute, Eulji University, Uijeongbu‑si, Gyeonggi‑Do Republic of Korea; 3 Department of Biological Sciences, Ulsan National Institute of Science and Technology, Ulsan, Republic of Korea; 4 Hematology Department, Eulji Medical Center, Eulji University, Uijeongbu‑si, Gyeonggi‑Do Republic of Korea; 5 Graduate School of Stem Cell and Regenerative Biology, Korea Advanced Institute of Science and Technology, Daejeon, Republic of Korea

**Keywords:** Chronic myeloid leukaemia, Oncogenes, Cell signalling, Acid, base, fluid, electrolyte disorders

Correction to: *Leukemia* (2026) 40:955–969; https://doi.org/10.1038/s41375-026-02916-3, published online 30 March 2026

Following the publication of this article, the authors noted an error in Figure 6A.

Specifically, for the RPS6 immunoblot corresponding to the K562 + asciminib condition, an image of the K562 + DMSO sample acquired at a different exposure time was inadvertently selected and incorporated in place of the correct K562 + asciminib image.

The incorrect Figure 6A is included below.

**Figure:**
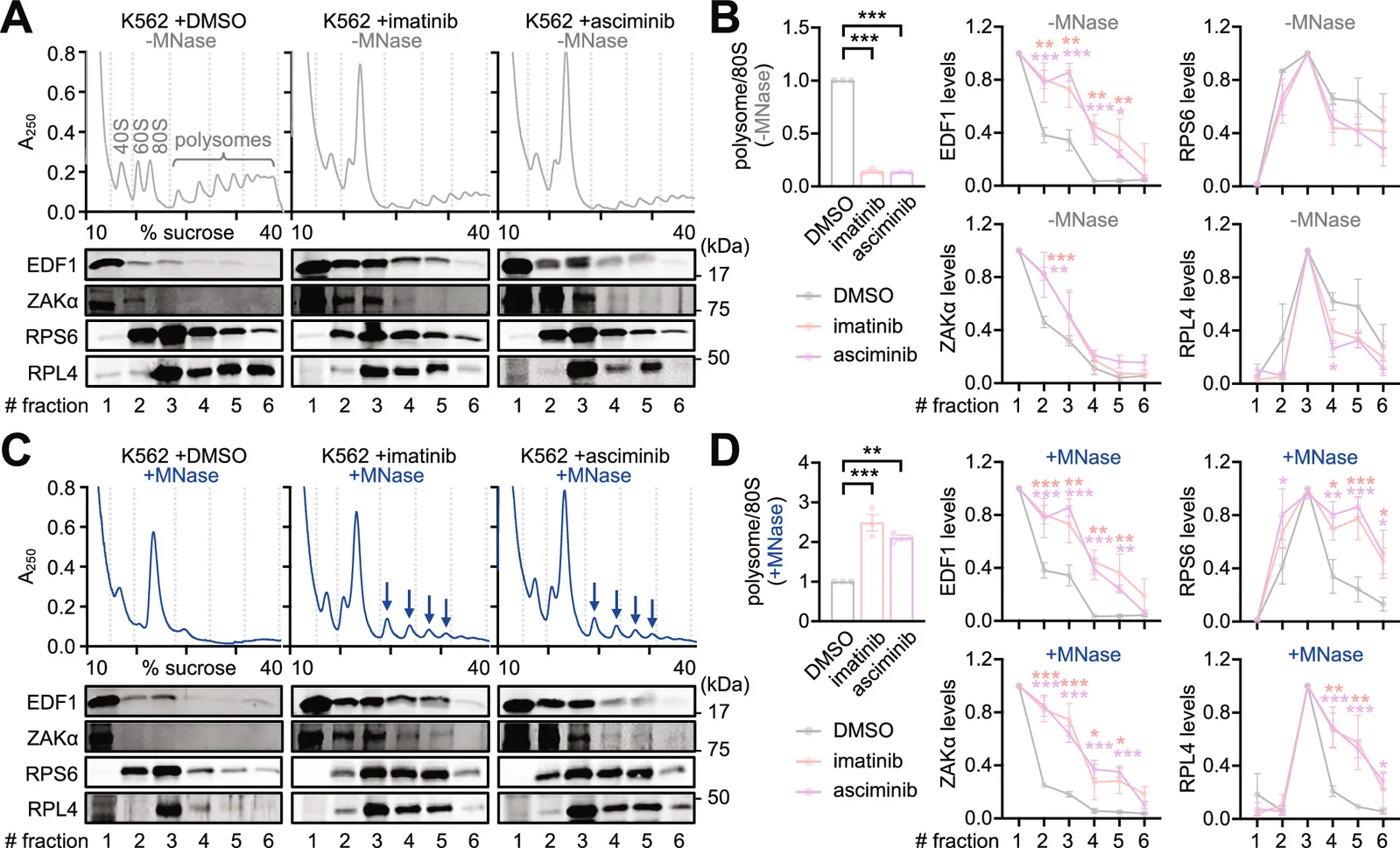

The corrected Figure 6A is included below.

**Figure:**
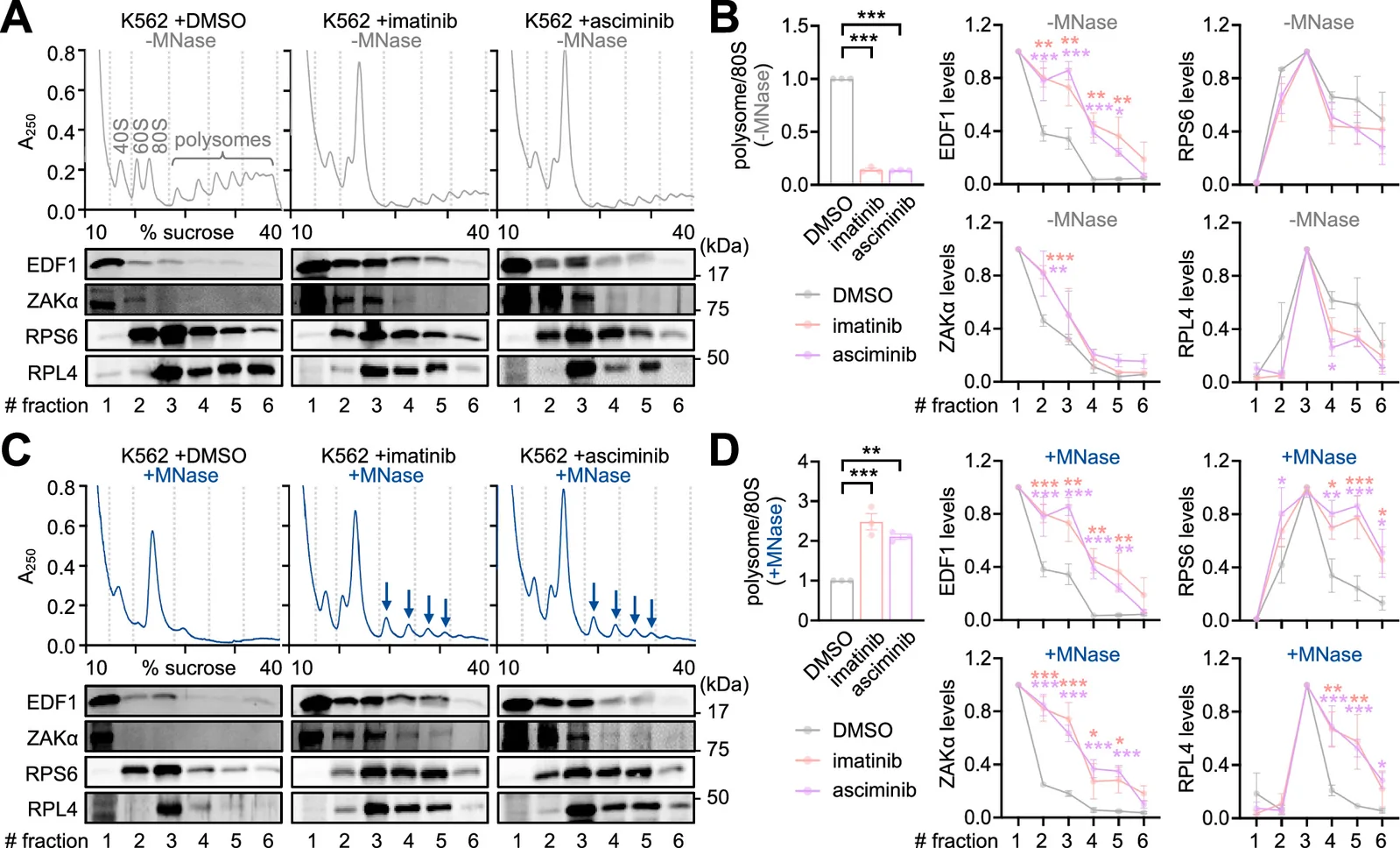

Importantly, the error was limited to the selection and assembly of the representative image. The quantitative results were generated from the correct original experimental data and are therefore unaffected. The statistical analyses and conclusions of the study also remain unchanged. The authors apologise for any inconvenience caused. The original article has been corrected.

