# Supplementary material for: BCR::ABL1 tyrosine kinase inhibitors induce ribosome collisions to activate ZAK-dependent ribotoxic stress and apoptosis in chronic myeloid leukemia
Source: Leukemia. 2026 Mar 30;40(5):955–69. doi: 10.1038/s41375-026-02916-3 (PMC13149316; doi:10.1038/s41375-026-02916-3)
Supplement: Supplementary file 1 — Supplementary Figures S1-S12 [file 41375_2026_2916_MOESM1_ESM.pdf]

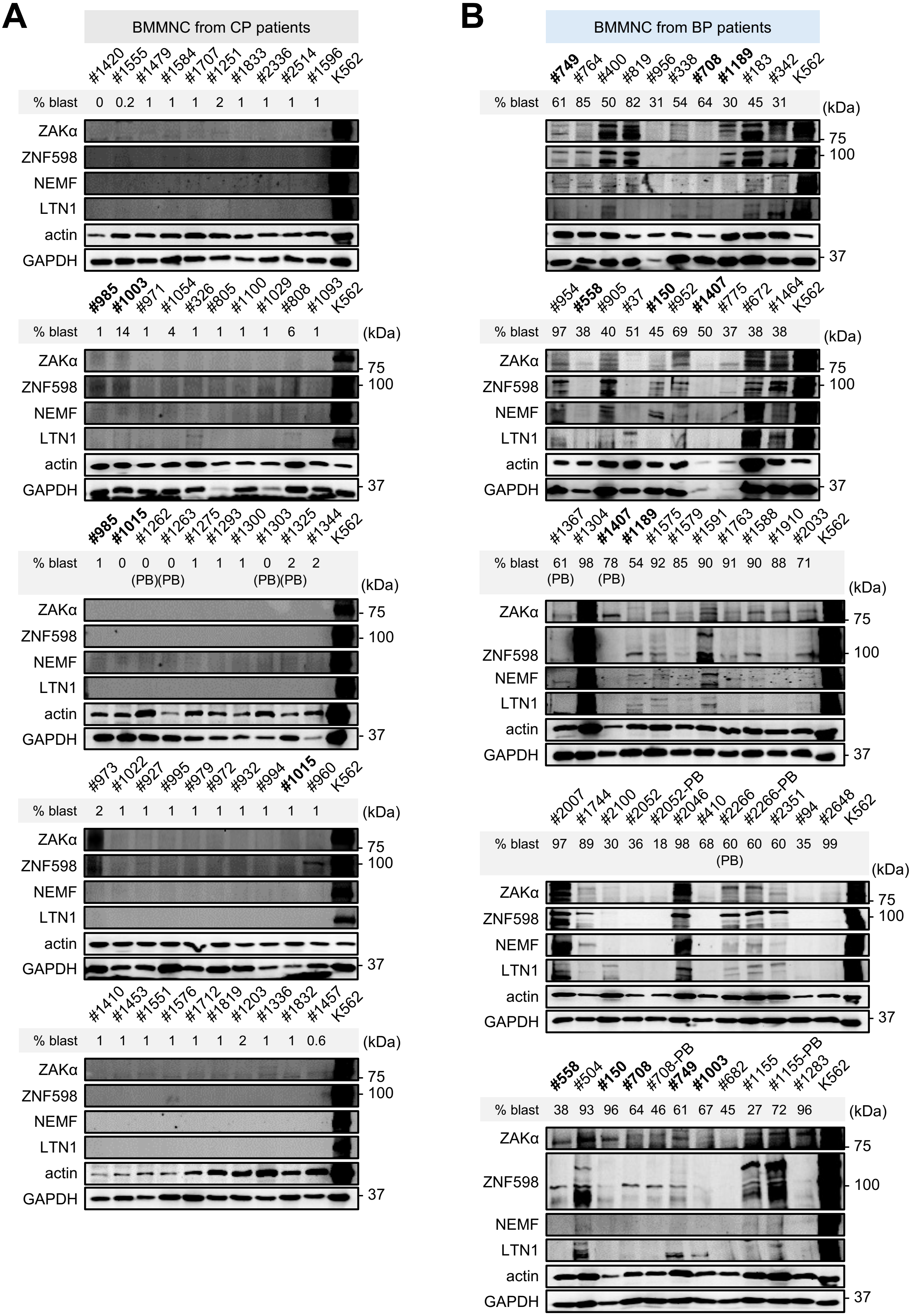
A
BMMNC from CP patients
#1420 #1555 #1479 #1584 #1707 #1251 #1833 #2336 #2514 #1596 K562
% blast 0 0.2 1 1 1 2 1 1 1 1
ZAKα
ZNF598
NEMF
LTN1
actin
GAPDH
#985 #1003 #971 #1054 #326 #805 #1100 #1029 #808 #1093 K562
% blast 1 14 1 4 1 1 1 1 6 1
(kDa)
75
100
37
#985 #1015 #1262 #1263 #1275 #1293 #1300 #1303 #1325 #1344 K562
% blast 1 0 0 0 1 1 1 0 2 2
(PB)(PB) (PB)(PB)
#973 #1022 #927 #995 #979 #972 #932 #994 #1015 #960 K562
% blast 2 1 1 1 1 1 1 1 1 1
#1410 #1453 #1551 #1576 #1712 #1819 #1203 #1336 #1832 #1457 K562
% blast 1 1 1 1 1 1 2 1 1 0.6
B
BMMNC from BP patients
#749 #764 #400 #819 #956 #338 #708 #1189 #183 #342 K562
% blast 61 85 50 82 31 54 64 30 45 31
#954 #558 #905 #37 #150 #952 #1407 #775 #672 #1464 K562
% blast 97 38 40 51 45 69 50 37 38 38
#1367 #1304 #1407 #1189 #1575 #1579 #1591 #1763 #1588 #1910 #2033 K562
% blast 61 98 78 54 92 85 90 91 90 88 71
(PB) (PB)
#2007 #1744 #2100 #2052 #2052-PB #2046 #410 #2266 #2266-PB #2351 #94 #2648 K562
% blast 97 89 30 36 18 98 68 60 60 60 35 99
(PB)
#558 #504 #150 #708 #708-PB #749 #1003 #682 #1155 #1155-PB #1283 K562
% blast 38 93 96 64 46 61 67 45 27 72 96

C

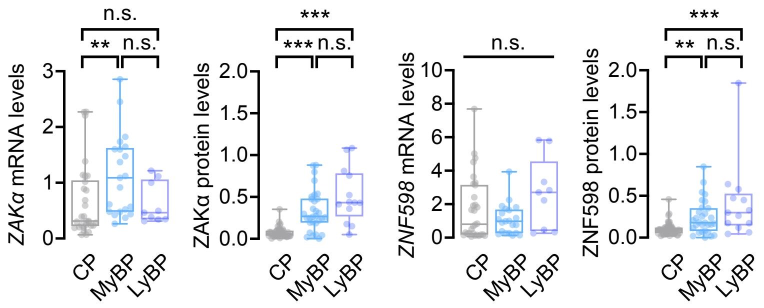

Figure S1. ZAKα and ZNF598 protein levels are upregulated in BP patient-derived BMMNCs. A, B Total BMMNC lysates from individual CML patients (A, CP = 48 patients; B, BP = 51 patients; also see Fig. 1D) were immunoblotted with the indicated antibodies. K562 cell lysates served as an internal control among independent immunoblottings. Patient ID and % blast cells were shown at the top. Bold patient ID, duplicated or independent samplings of identical patients; PB, peripheral blood samples. **C** BP patients display high ZAK expression with lineage-associated differences at transcript but not protein levels. ZAKα and ZNF598 expression were quantified in BMMNCs from CML patients [CP = 30 patients, myeloid BP (MyBP) = 21 patients, and lymphoid BP (LyBP) = 9 patients for quantitative PCR analysis; CP = 48 patients, MyBP = 30 patients, and LyBP = 13 patients for immunoblotting analysis]. The relative abundance of each transcript and protein was quantified as in Fig. 1D. n.s., not significant; $^{**}P < 0.01$, $^{***}P < 0.001$ as determined by ART 1-way ANOVA, Wilcoxon rank sum test.

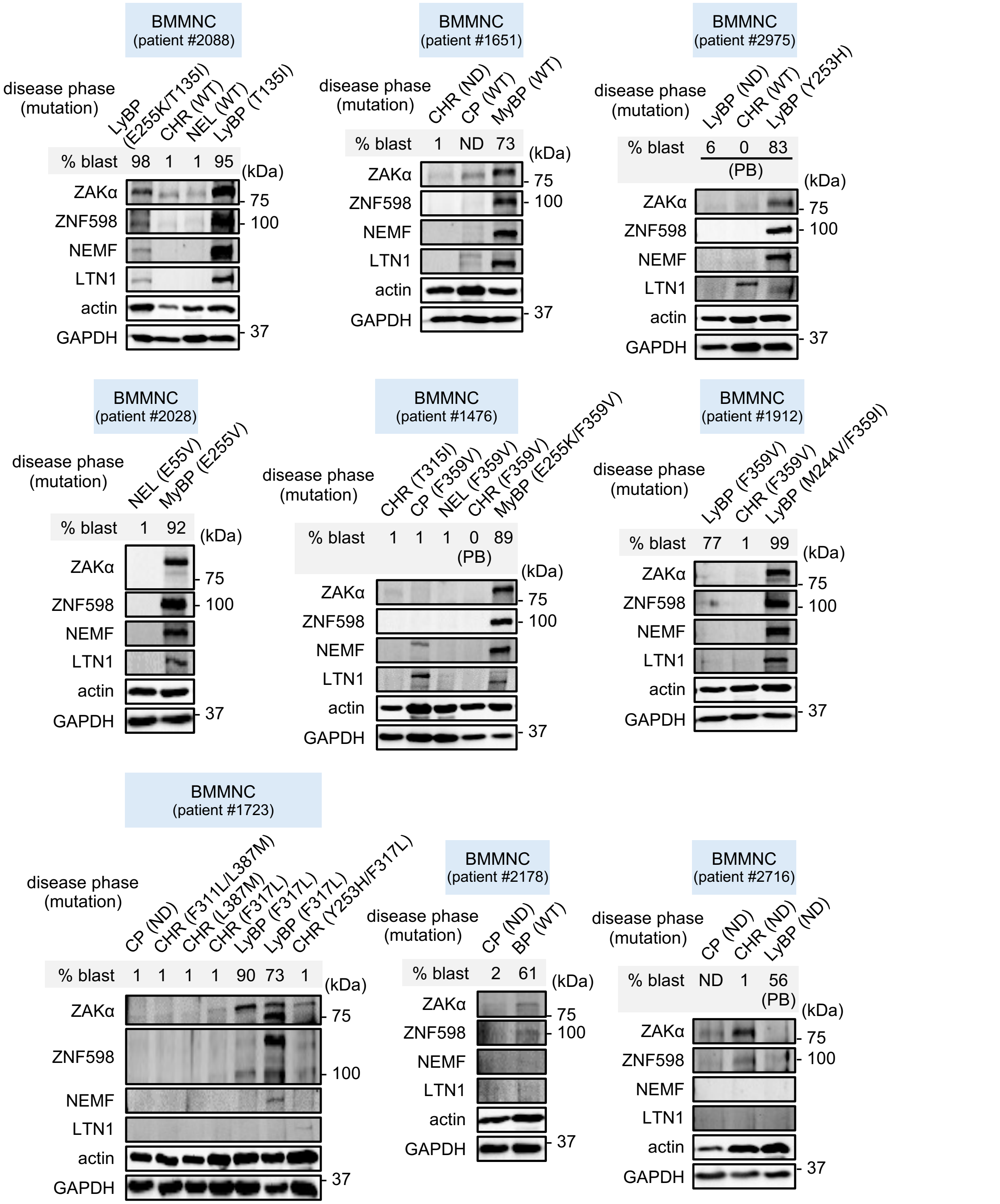

Figure S2. BP progression in each CML patient accompanies elevated expression of ZAKα protein and a subset of RQC factors. BMMNC samples were serially obtained from individual patients at different CML stages (*n* = 10 patients; also see Fig. 1E). Protein expression was assessed by immunoblotting of total cell lysates. Patient ID and % blast were shown at the top. PB, peripheral blood samples. Specific BCR::ABL1 mutations detected were given in parentheses. WT, wild-type; ND, not determined; CP, chronic phase; BP, blast phase; CHR, complete hematologic response; NEL, no evidence of leukemia; MyBP, myeloid blast phase; LyBP, lymphoid blast phase.

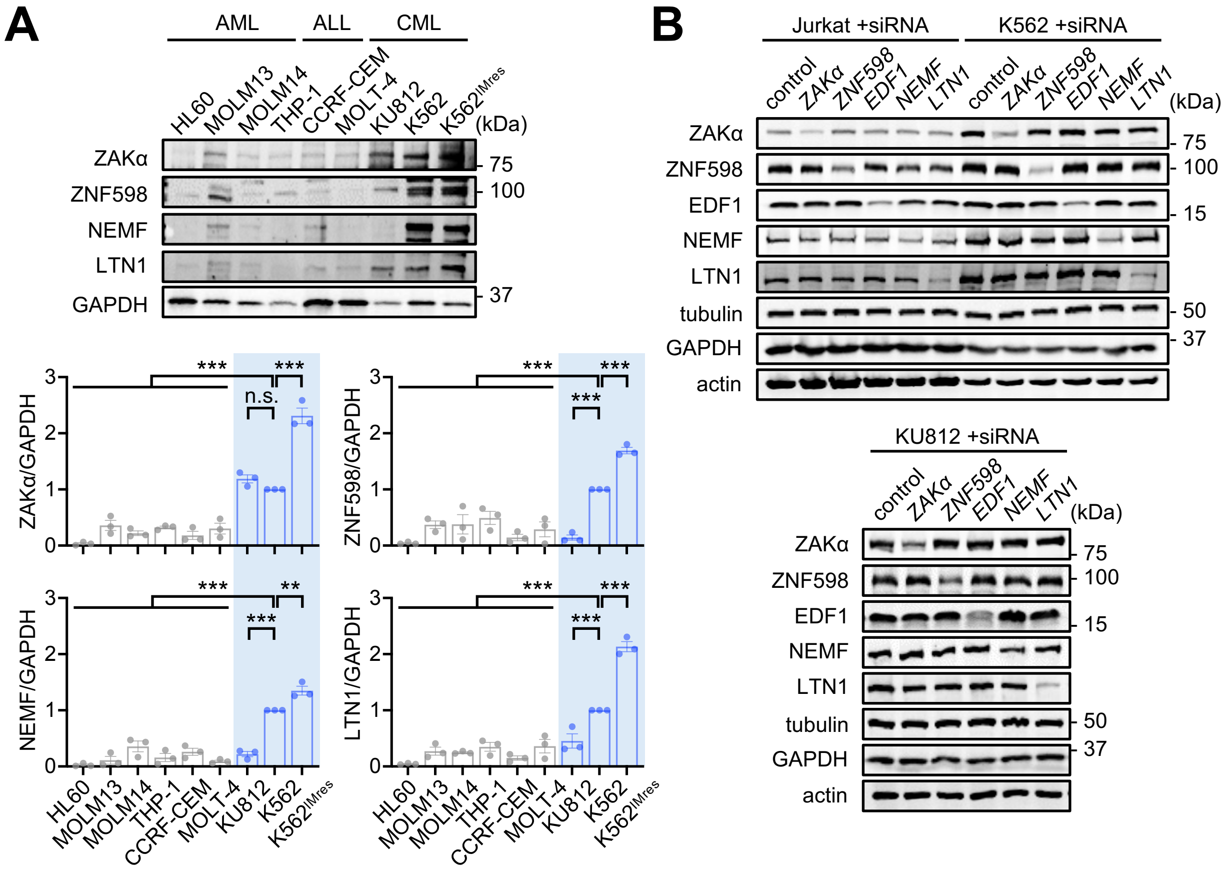

Figure S3. ZAKα and a subset of RQC factors are upregulated in CML cell lines. A CML cell lines express relatively high levels of ZAK and a subset of RQC factors (ZNF598, NEMF, and LTN1). Total cell lysates were prepared from various leukemia cell lines (AML, acute myeloid leukemia; ALL, acute lymphocytic leukemia; CML, chronic myelogenous leukemia; K562^{IMres}, an imatinib-resistant K562 cell line) and immunoblotted with the indicated antibodies. Protein expression was quantified as in Fig. 1D. Data represent mean +/- SEM (n = 3). n.s., not significant; $^{**}P < 0.01$, $^{***}P < 0.001$ as determined by 1-way ANOVA, Dunnett's multiple comparisons test. **B** Transient transfection with individual siRNAs targeting ZAK or RQC factors depletes the corresponding proteins comparably among non-CML (Jurkat) and CML cell lines (K562 and KU812). Total cell lysates were prepared 48 h after siRNA transfection and immunoblotted with the indicated antibodies.

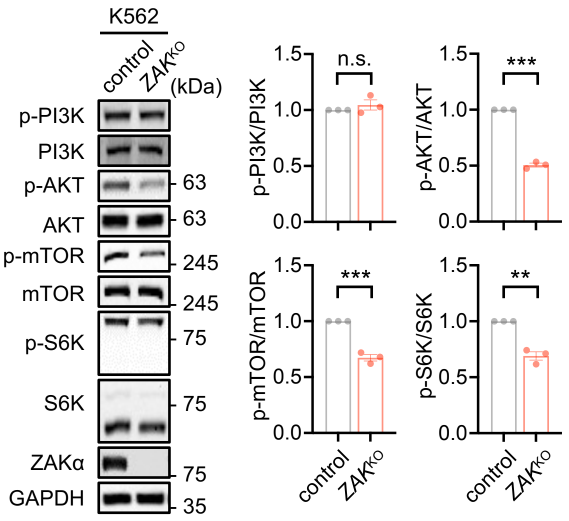

Figure S4. *ZAK* deletion attenuates AKT-mTOR signaling in K562 cells. Total cell lysates were prepared from control and *ZAK*-deleted K562 cells (ZAK^{KO}) and subjected to immunoblot analysis using the indicated antibodies. Protein expression was quantified by the corresponding band intensity in immunoblotting. The relative abundance of phosphorylated proteins was calculated by normalizing the ratio of phospho-specific to total signals to that in control (set as 1). Data represent mean +/- SEM (n = 3). n.s., not significant; **$P < 0.01$, ***$P < 0.001$ as determined by unpaired t-test.

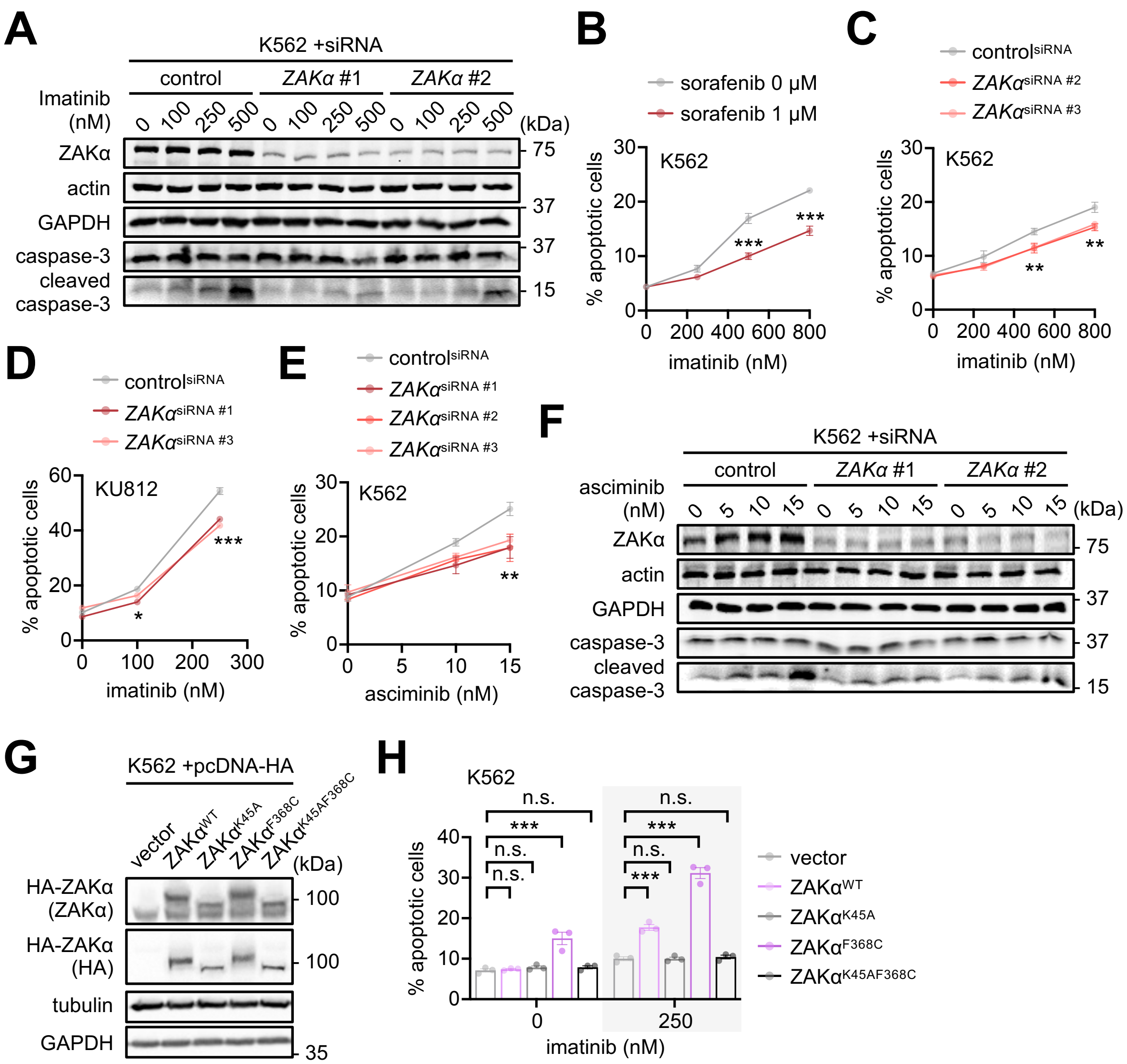
A
K562 +siRNA
control
ZAKα #1
ZAKα #2
Imatinib (nM)
0 100 250 500 0 100 250 500 0 100 250 500
(kDa)
ZAKα
actin
GAPDH
caspase-3
cleaved caspase-3
75
37
37
15
B
sorafenib 0 μM
sorafenib 1 μM
K562
% apoptotic cells
imatinib (nM)
C
control siRNA
ZAKα siRNA #2
ZAKα siRNA #3
K562
% apoptotic cells
imatinib (nM)
D
control siRNA
ZAKα siRNA #1
ZAKα siRNA #3
KU812
% apoptotic cells
imatinib (nM)
E
control siRNA
ZAKα siRNA #1
ZAKα siRNA #2
ZAKα siRNA #3
K562
% apoptotic cells
asciminib (nM)
F
K562 +siRNA
control
ZAKα #1
ZAKα #2
asciminib (nM)
0 5 10 15 0 5 10 15 0 5 10 15
(kDa)
ZAKα
actin
GAPDH
caspase-3
cleaved caspase-3
75
37
37
15
G
K562 +pcDNA-HA
vector
ZAKα WT
ZAKα K45A
ZAKα F368C
ZAKα K45AF368C
(kDa)
HA-ZAKα (ZAKα)
HA-ZAKα (HA)
tubulin
GAPDH
100
100
35
H
K562
n.s.
***
% apoptotic cells
imatinib (nM)
vector
ZAKα WT
ZAKα K45A
ZAKα F368C
ZAKα K45AF368C

Figure S5. ZAK mediates CML apoptosis upon BCR::ABL1 inhibition. A ZAK depletion suppresses imatinib-induced caspase-3 cleavage in K562 cells. Total cell lysates were prepared 48 h after imatinib treatment of siRNA-transfected cells. Protein expression was analyzed by immunoblotting with the indicated antibodies. **B** Pharmacological ZAK inhibition reduces imatinib-induced apoptosis in K562 cells. K562 cells were co-treated with sorafenib and imatinib 48 h before flow cytometry analysis. The percentage of apoptotic cells was scored as in Fig. 3B. Two-way ANOVA detected significant interaction effects between sorafenib and imatinib on % apoptotic cells ($P < 0.0001$). Data represent mean +/- SEM ($n = 3$). ***$P < 0.001$ to vehicle control (sorafenib 0 μM = DMSO) at given imatinib concentrations, as determined by Tukey's multiple comparisons test. **C,D** ZAK depletion suppresses imatinib-induced cell death in K562 or KU812 cells. siRNA-transfected cells were incubated with imatinib for 48 h before flow cytometry analysis. Two-way ANOVA detected significant effects of ZAK on % apoptotic cells in K562 cells ($P = 0.0116$ for siRNA #2; $P = 0.0110$ for siRNA #3) and in KU812 cells ($P < 0.001$ for both siRNAs). Data represent mean +/- SEM ($n = 3$). *$P < 0.05$, **$P < 0.01$, ***$P < 0.001$ to control siRNA at given imatinib concentrations, as determined by Tukey's multiple comparisons test. **E** ZAK depletion reduces asciminib-induced apoptosis in K562 cells. Two-way ANOVA detected significant effects of ZAK ($P = 0.0037$ for siRNA #1; $P = 0.0047$ for siRNA #2; $P = 0.0101$ for siRNA #3) on % apoptotic cells. Data represent mean +/- SEM ($n = 3$). **$P < 0.01$ to control siRNA at given asciminib concentrations, as determined by Tukey's multiple comparisons test. **F** ZAK depletion reduces asciminib-induced caspase-3 cleavage in K562 cells. Total cell lysates were prepared 48 h after asciminib treatment of siRNA-transfected cells. Protein expression was analyzed by immunoblotting with the indicated antibodies. **G** Transgenic expression of wild-type and mutant ZAK proteins in K562 cells. Total cell lysates were prepared 48 h after transfection with expression vectors for HA-tagged ZAK and immunoblotted with the indicated antibodies. **H** ZAK overexpression promotes imatinib-induced CML apoptosis in a kinase activity-dependent manner. K562 cells were transfected with expression vectors for wild-type (WT) or mutant ZAK proteins (K45A, kinase-dead; F368C, constitutively active; K45AF368C, double mutant). At 48 h posttransfection, cells were treated with imatinib and further incubated for 48 h before flow cytometry analysis ($n = 50,000$ cells). Two-way ANOVA detected significant effects of ZAK^{WT} ($P < 0.0001$) and ZAK^{F368C} ($P = 0.8637$), but not ZAK^{K45A} ($P = 0.3968$) and $ZAK^{K45AF368C}$ ($P = 0.6916$), on % apoptotic cells. Data represent mean +/- SEM ($n = 3$). n.s., not significant; ***$P < 0.001$ to vector controls at given imatinib concentrations, as determined by Tukey's multiple comparisons test.

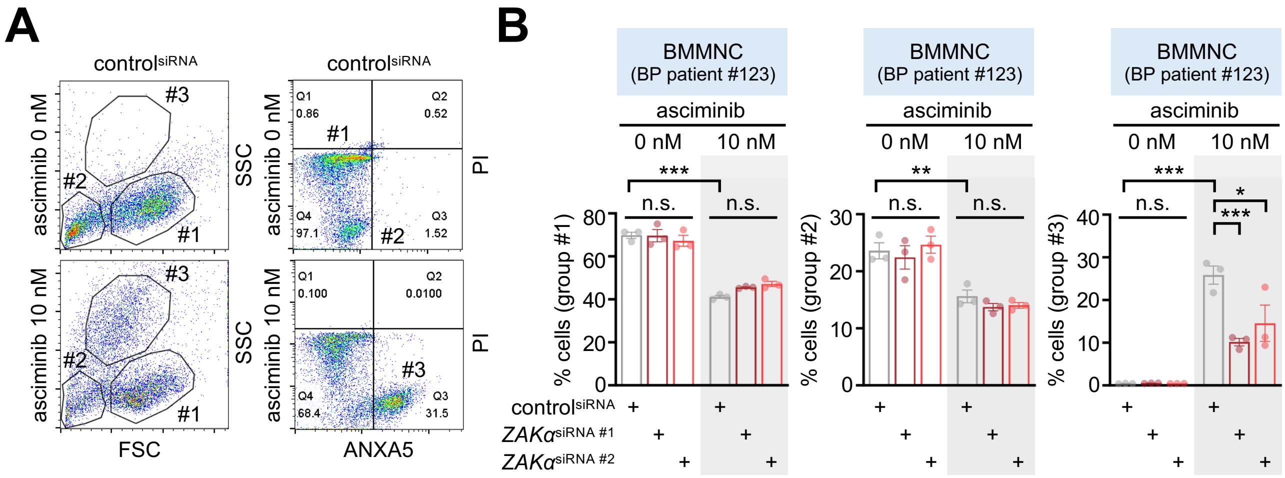

Figure S6. ZAK depletion suppresses asciminib-induced apoptosis in CML patient cells. A BP patient-derived BMMNCs exhibited two distinct populations (groups #1 and #2) as revealed by forward scatter (FSC) and side scatter (SSC). Asciminib treatment of the primary BP cell cultures generated an apoptotic cell population (group #3). **B** ZAK depletion suppresses asciminib-induced apoptosis in CML patient-derived BMMNCs. BP patient-derived BMMNCs were transfected with the indicated siRNAs. At 48 h posttransfection, cells were treated with asciminib and further incubated for 48 h before flow cytometry analysis (*n* = 10,000 cells). The percentage of cells present in each gated area (groups #1, #2, and #3) was measured in relation to the intensity of FITC–Annexin V (ANXA5) and propidium iodide (PI) staining. Two-way ANOVA detected significant interaction effects between ZAK and asciminib on % cells present in group #3 ($P = 0.0001$ for siRNA #1; $P = 0.0447$ for siRNA #2). Data represent mean +/- SEM ($n = 3$). n.s., not significant; $^{*}P < 0.05$, $^{**}P < 0.01$, $^{***}P < 0.001$ as determined by Tukey's multiple comparisons test.

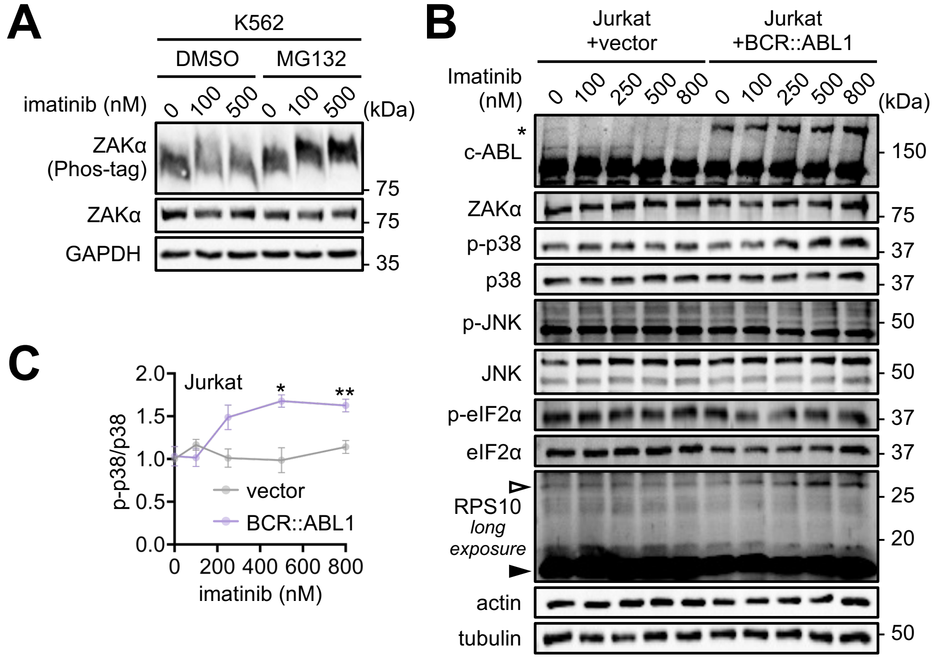

Figure S7. BCR::ABL1 inhibition induces ZAKα and p38 phosphorylation, as well as RPS10 ubiquitination. A Imatinib treatment of K562 cells induces endogenous ZAKα phosphorylation. K562 cells were co-treated with imatinib and MG132 (1 μM) for 6 h before protein analysis. Total cell lysates were resolved in the Phos-tag gel for phosphorylation-sensitive separation and immunoblotted with the indicated antibodies. **B, C** Imatinib treatment promotes p38 phosphorylation and RPS10 ubiquitination in BCR::ABL1-overexpressing Jurkat cells but not in control Jurkat cells. Jurkat cells were transfected with expression vector for BCR::ABL1 protein. At 48 h post-transfection, cells were treated with imatinib and further incubated for 6 h before harvest. Total cell lysates were immunoblotted using the indicated antibodies. Data represent mean +/- SEM (n = 3). *$P < 0.05$, **$P < 0.01$ to vector control at given imatinib concentrations, as determined by Student t test. Asterisk, BCR::ABL1; white arrowhead, ubiquitinated RPS10; black arrowhead, RPS10.

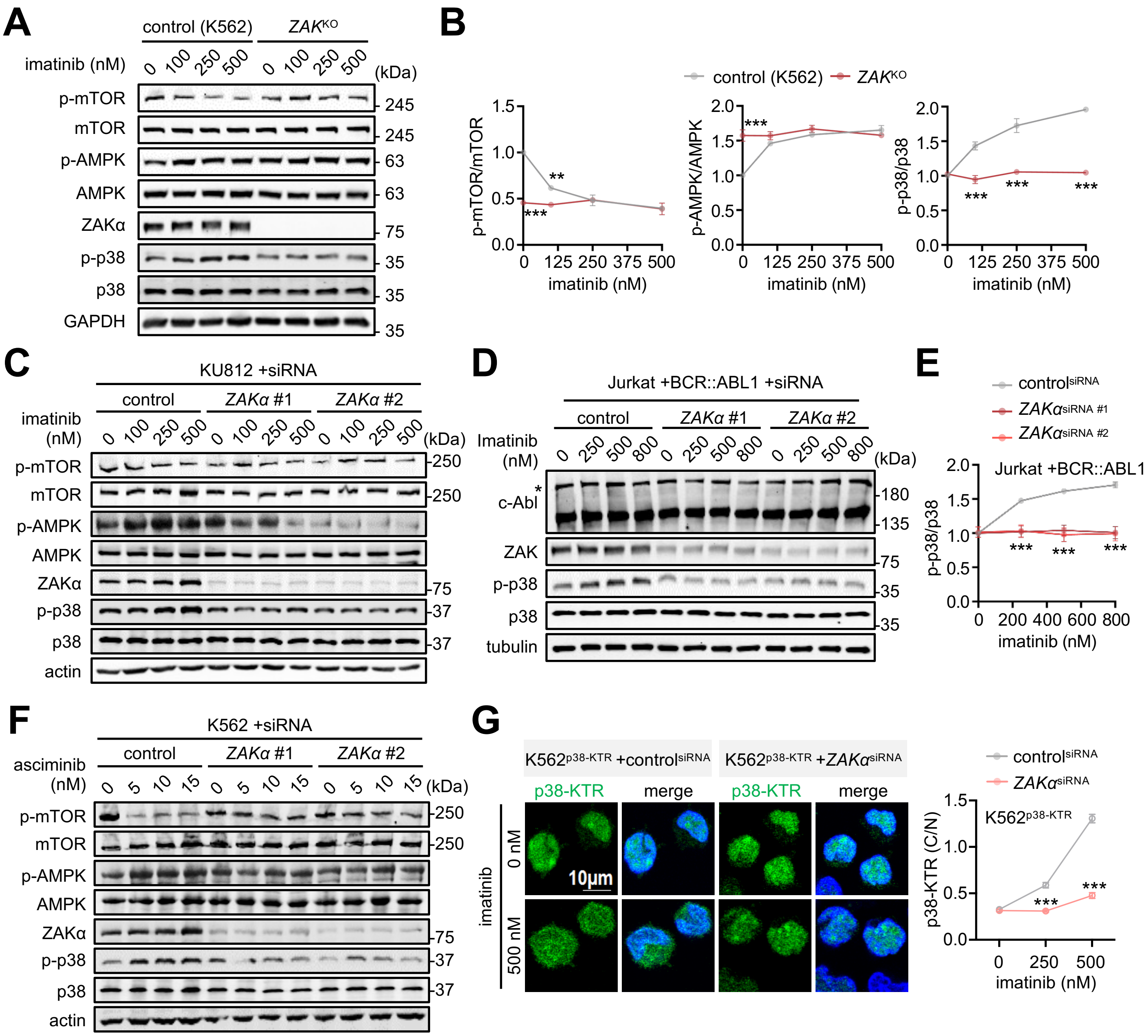
A
control (K562)
ZAK^KO
imatinib (nM)
0 100 250 500 0 100 250 500
(kDa)
p-mTOR
mTOR
p-AMPK
AMPK
ZAKα
p-p38
p38
GAPDH
245
245
63
63
75
35
35
35
B
control (K562)
ZAK^KO
p-mTOR/mTOR
p-AMPK/AMPK
p-p38/p38
imatinib (nM)
0 125 250 375 500
***
**
C
KU812 +siRNA
control
ZAKα #1
ZAKα #2
imatinib (nM)
0 100 250 500
(kDa)
p-mTOR
mTOR
p-AMPK
AMPK
ZAKα
p-p38
p38
actin
250
250
75
37
37
D
Jurkat +BCR::ABL1 +siRNA
control
ZAKα #1
ZAKα #2
Imatinib (nM)
0 250 500 800
(kDa)
c-Abl
*
ZAK
p-p38
p38
tubulin
180
135
75
35
35
E
control^siRNA
ZAKα^siRNA #1
ZAKα^siRNA #2
Jurkat +BCR::ABL1
p-p38/p38
0 200 400 600 800
imatinib (nM)
***
F
K562 +siRNA
control
ZAKα #1
ZAKα #2
asciminib (nM)
0 5 10 15
(kDa)
p-mTOR
mTOR
p-AMPK
AMPK
ZAKα
p-p38
p38
actin
250
250
75
37
37
G
K562^p38-KTR +control^siRNA
K562^p38-KTR +ZAKα^siRNA
p38-KTR
merge
imatinib
0 nM
500 nM
10μm
control^siRNA
ZAKα^siRNA
K562^p38-KTR
p38-KTR (C/N)
0 250 500
imatinib (nM)
***

Figure S8. BCR::ABL1 inhibition induces ZAK-dependent RSR. A, B *ZAK* deletion abolishes imatinib-induced ribotoxic stress responses in K562 cells. Control (K562) and ZAK^{KO} cells were incubated with imatinib for 6 h before protein analyses. Protein expression was quantified by the corresponding band intensity in immunoblotting. The relative abundance of phosphorylated proteins was calculated by normalizing the ratio of phospho-specific to total signals to that in control (0 nM imatinib; set as 1). Two-way ANOVA detected significant interaction effects between *ZAK* deletion and imatinib treatment on mTOR, AMPK, and p38 phosphorylation ($P < 0.0001$). Data represent mean +/- SEM ($n = 3$). **$P < 0.01$, ***$P < 0.001$ to control at given imatinib concentrations, as determined by Tukey's multiple comparisons test. **C, F** ZAK depletion blunts phosphorylation-dependent responses of p38, mTOR, and AMPK to BCR-ABL inhibition in CML cell lines. siRNA-transfected cells were incubated with imatinib for 6 h (C) or asciminib for 12 h (F) before protein analyses. Total cell lysates were immunoblotted using the indicated antibodies. **D, E** ZAK depletion abolishes imatinib-induced p38 phosphorylation in BCR::ABL1-overexpressing Jurkat cells. Jurkat cells were co-transfected with expression vector for BCR::ABL1 protein and siRNAs. At 48 h post-transfection, cells were treated with imatinib and further incubated for 6 h before harvest. Two-way ANOVA detected significant interaction effects between ZAK depletion and imatinib treatment on p38 phosphorylation ($P < 0.0001$ for siRNA #1; $P = 0.0003$ for siRNA #2). Data represent mean +/- SEM ($n = 3$). ***$P < 0.001$ to control siRNA at given imatinib concentrations, as determined by Tukey's multiple comparisons test. **G** Imatinib treatment induces cytoplasmic translocation of the fluorescence reporter for p38 phosphorylation in a ZAK-dependent manner. K562 cells stably expressing the p38-KTR sensor ($K562^{p38\text{-}KTR}$) were transfected with control or *ZAK* siRNAs. At 48 h after transfection, cells were incubated with imatinib for 6 h before Hoechst 33258 staining (blue, nucleus). The p38-KTR reporter activation was quantified by calculating the intensity ratio of cytoplasmic to nuclear (C/N) fluorescence in individual cells. Data represent means +/- SEM ($n = 50$ cells). ***$P < 0.001$ to control siRNA at given imatinib concentrations, as determined by 2-way ANOVA, Tukey's multiple comparisons test.

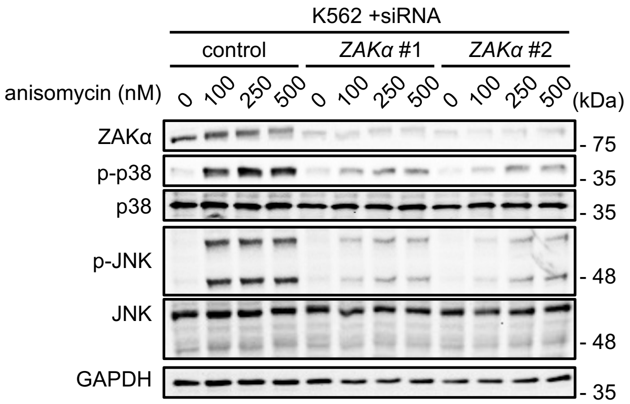

Figure S9. Anisomycin treatment induces ZAK-dependent phosphorylation of JNK and p38 in K562 cells. siRNA-transfected K562 cells were incubated with anisomycin for 30 min before immunoblotting of total cell lysates with the indicated antibodies.

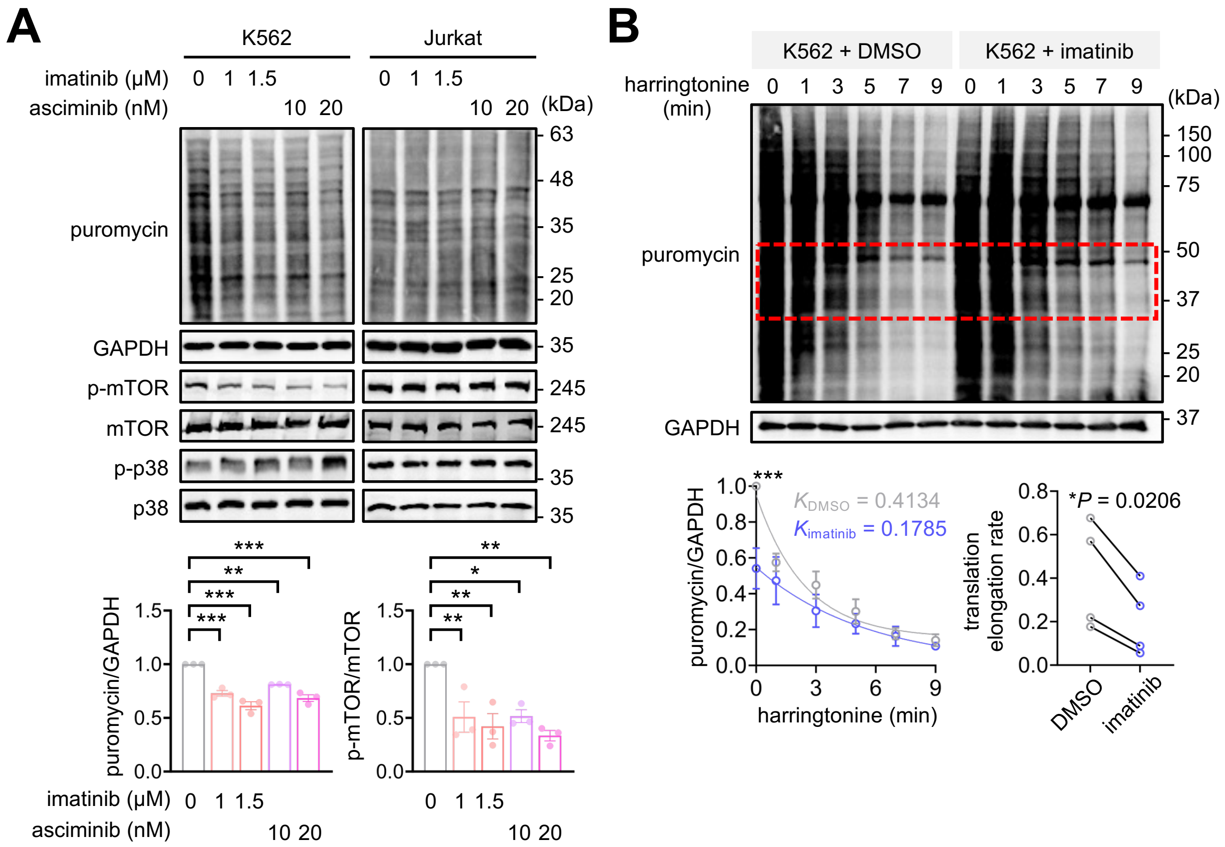

Figure S10. BCR::ABL1 inhibition slows down translation elongation. A BCR::ABL1 inhibitor reduces the relative levels of puromycin-labeled nascent polypeptides in BCR::ABL1-positive K562 but not in BCR::ABL1-negative Jurkat cells. K562 and Jurkat cells were treated with the BCR::ABL1 inhibitors imatinib or asciminib for 6 h before puromycin labeling (10 µg/ml for 10 min). Total cell lysates were immunoblotted with the indicated antibodies. The relative abundance of puromycin-labeled (bottom, left) or mTOR phosphorylation (bottom, right) in K562 cells was calculated by normalizing to vehicle control (DMSO-treated cells; set as 1). Data represent mean +/- SEM (n = 3). *P < 0.05, **P < 0.01, ***P < 0.001 as determined by 1-way ANOVA, Dunnett's multiple comparisons test. **B** K562 cells were treated with 1 µM imatinib or vehicle control (DMSO) for 6 h before harringtonine run-off of translating ribosomes. Imatinib-treated cells were incubated with 2 µM harringtonine for the indicated time and then labeled with 10 µg/ml puromycin for 10 min. Total cell lysates were analyzed by immunoblotting with anti-puromycin (top) and anti-GAPDH (bottom) antibodies. Puromycin-labeled nascent polypeptides were quantified by measuring the intensity of 50–37 kDa protein bands (red-dotted box). The relative abundance of puromycin-labeled proteins was calculated by normalizing to the DMSO control at 0 min of harringtonine treatment (set as 1) and fit to a one-phase decay to obtain a rate constant as a proxy for translation elongation rate. Data represent means +/- SEM (n = 4). *P < 0.05, ***P < 0.001 as determined by 2-way ANOVA, Tukey's multiple comparisons test (puromycin levels) or paired t-test (translation elongation rate).

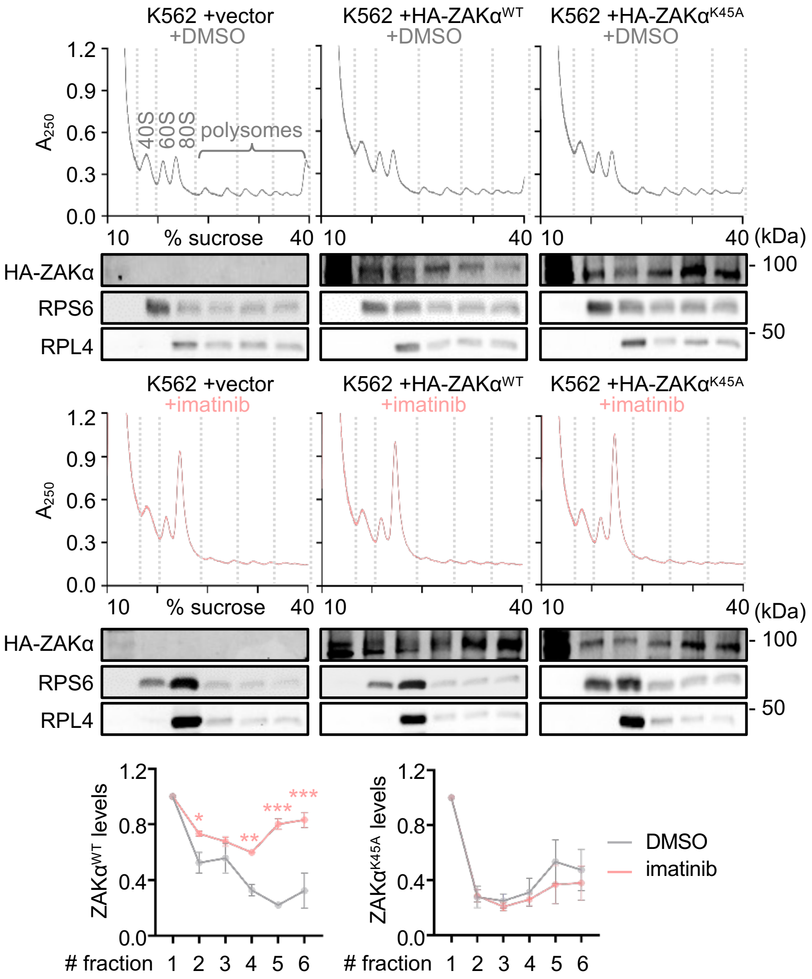

Figure S11. BCR::ABL1 inhibition promotes polysomal association of ZAK in a manner dependent on ZAK kinase activity. K562 cells transfected with ZAKα expression vectors (HA-ZAKα^{WT} or HA-ZAKα^{K45A}) were treated with 1 µM imatinib or vehicle control (DMSO) for 6 h before harvest. Soluble cell lysates were loaded onto a 10-40% sucrose density gradient for biochemical fractionation by ultracentrifugation. Absorbance at 250 nm was continuously monitored during fraction collection. Protein samples were precipitated from each fraction (dotted lines) and analyzed by immunoblotting. Relative protein levels in individual fractions were calculated by normalizing to the peak fraction per condition (set as 1). Data represent mean +/- SEM (n = 3). *$P < 0.05$, **$P < 0.01$, ***$P < 0.001$ to the vehicle control in each fraction, as determined by 2-way ANOVA, Tukey's multiple comparisons test.

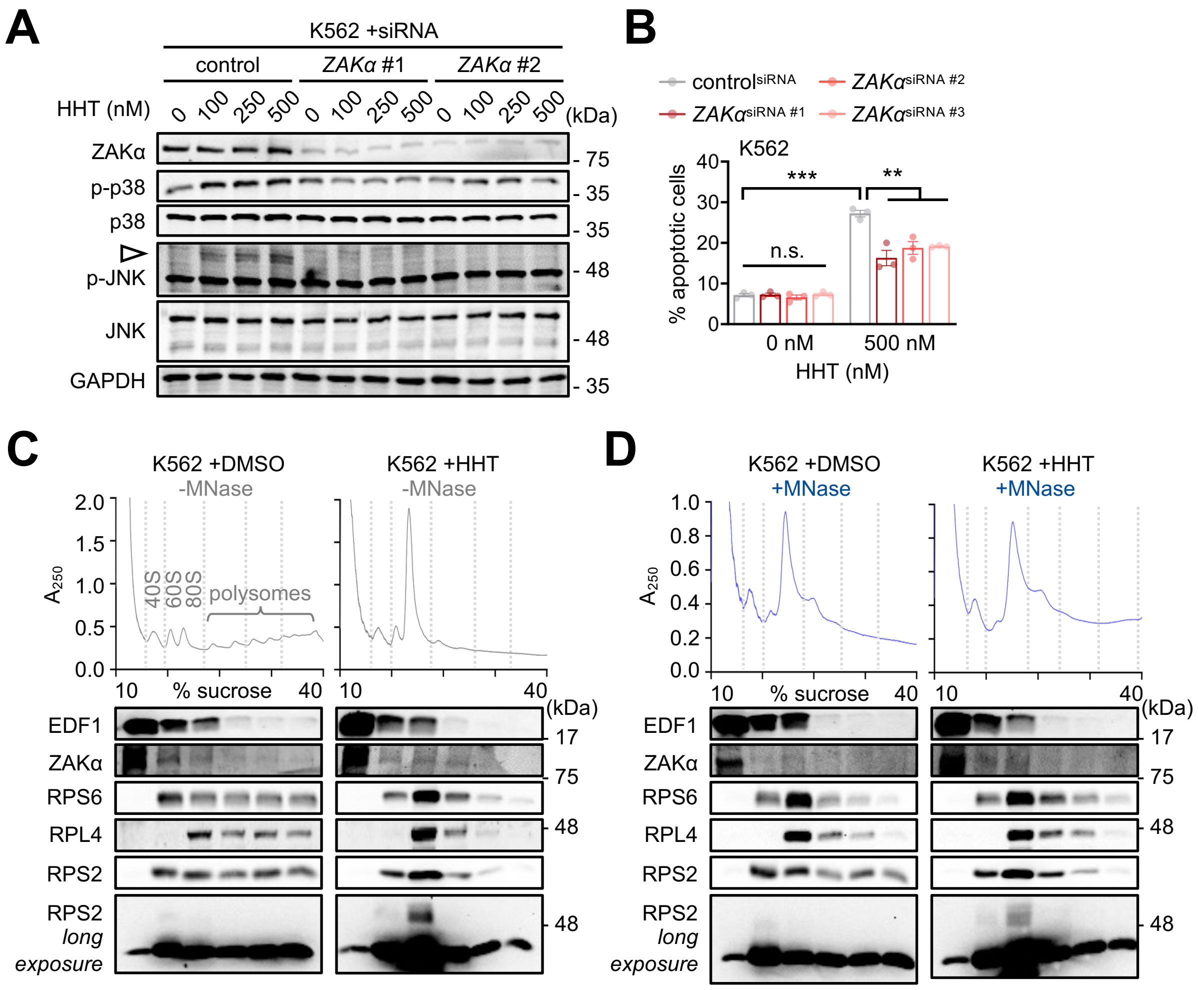

Figure S12. Homoharringtonine (HTT) induces ribosome pausing at translation initiation to activate ZAK-dependent RSR in K562 cells. A HHT treatment induces ZAK-dependent SAPK phosphorylation in K562 cells. siRNA-transfected cells were incubated with HHT for 1 h before immunoblotting of total cell lysates with the indicated antibodies. White arrowhead, phosphorylated JNK. **B** ZAK depletion suppresses HHT-induced apoptosis in K562 cells. siRNA-transfected cells were treated with HHT for 48 h before flow cytometry analysis of apoptotic cells. Two-way ANOVA detected significant interaction effects between ZAK depletion and HHT treatment on % apoptotic cells (P = 0.0008 for siRNA #1; P = 0.0032 for siRNA #2; $P < 0.0001$ for siRNA #3). Data represent mean +/- SEM (n = 3). n.s., not significant; **$P < 0.01$, ***$P < 0.001$ as determined by Tukey's multiple comparisons test. **C** HHT treatment reduces the polysome-to-monosome (80S) ratio but does not shift the ribosome collision sensors EDF1 and ZAK into polysomal fractions. K562 cells were treated with HHT (500 nM) or DMSO (vehicle control) for 1 h before harvest. Soluble cell lysates were loaded onto a 10-40% sucrose density gradient for biochemical fractionation by ultracentrifugation. Absorbance at 250 nm was continuously monitored during fraction collection. Protein samples were precipitated from each fraction (dotted lines) and analyzed by immunoblotting. **D** HHT treatment does not generate nuclease-resistant polysomes in the sucrose-density gradient profile of ribosomal populations. Soluble cell lysates from HHT-treated K562 cells were incubated with micrococcal nuclease (MNase, 500 U) for 30 min to digest polysomal mRNAs into ribosome-protected ones before loading onto a 10-40% sucrose density gradient.
